# Oxygen scavenging enables microoxic survival of the marine anammox bacterium *Scalindua* sp

**DOI:** 10.1093/ismejo/wrag075

**Published:** 2026-04-02

**Authors:** Satoshi Okabe, Keishi Nukada, Keitaro Horiguchi, Mamoru Oshiki

**Affiliations:** Division of Environmental Engineering, Faculty of Engineering, Hokkaido University, North-13, West-8, Kita-ku, Sapporo, Hokkaido 060-8628, Japan; Division of Environmental Engineering, Graduate School of Engineering, Hokkaido University, North-13, West-8, Kita-ku, Sapporo, Hokkaido 060-8628, Japan; Division of Environmental Engineering, Graduate School of Engineering, Hokkaido University, North-13, West-8, Kita-ku, Sapporo, Hokkaido 060-8628, Japan; Division of Environmental Engineering, Faculty of Engineering, Hokkaido University, North-13, West-8, Kita-ku, Sapporo, Hokkaido 060-8628, Japan

**Keywords:** anammox, *Scalindua* sp, specific oxygen reduction rate (SORR), cytochrome *c* oxidase (CcO), ROS detoxification

## Abstract

Marine anammox bacteria of the genus *Scalindua* play a pivotal role in nitrogen loss within oceanic oxygen minimum zones (OMZs), yet their physiological resilience to oxygen exposure remains poorly characterized. Here, we show that *Scalindua* sp. possesses an intrinsic capacity to reduce ambient oxygen under microoxic conditions (headspace O₂ ≈ 3%), exhibiting specific oxygen reduction rates of ~10 nmol-O₂ min^−1^ mg- protein^−1^. Anammox activity, evidenced by ^29^N₂ production, resumed once dissolved oxygen (DO) declined below ~5 μM, defining the DO threshold. This oxygen-scavenging ability is likely mediated by direct oxygen reduction to water via cbb₃-type cytochrome *c* oxidase (CcO) and A-type flavodiiron proteins, with minimal reactive oxygen species (ROS) generation. CcO activity increased rapidly and markedly upon nitrite addition, consistent with the immediate onset of oxygen consumption and the transcriptional induction of CcO subunit genes. Nitrite was essential for sustaining oxygen consumption, and the rapid CcO activation suggests that nitrite stimulates CcO function through an unresolved regulatory mechanism. Nitrite oxidation also supplies supplementary electrons for oxygen reduction independently of core anammox metabolism, underscoring a flexible substrate-driven detoxification mechanism that enables *Scalindua* sp. to cope with transient microoxia. Together, these results show that *Scalindua* sp. employs a multi-layered defense—moderate oxygen-reduction capacity coupled with efficient ROS detoxification—conferring high and reversible oxygen tolerance. Although laboratory conditions cannot fully replicate natural OMZ complexity, our findings indicate that this physiological flexibility reflects the potential ecological resilience of *Scalindua* sp. in dynamic ocean environments.

## Introduction

Anaerobic ammonium oxidation (anammox) is a key microbial process in which ammonium (NH₄^+^) is oxidized under anoxic conditions using nitrite (NO₂^−^) as the electron acceptor, resulting in the direct production of dinitrogen gas (N₂). Together with denitrification, anammox contributes substantially to fixed nitrogen loss in marine ecosystems [1, 2]. In particular, within oxygen minimum zones (OMZs)—regions where dissolved oxygen (DO) concentrations fall below 20 μM—anammox are estimated to account for ~20%–70% of total oceanic nitrogen loss [3, 4, 5, 6, 7].

Anammox bacteria are obligate anaerobes ubiquitously distributed across low-oxygen natural and engineered environments [8, 9]. Ambient oxygen concentration is widely recognized as the primary factor constraining their ecological niches [10, 11]. Among known taxa, *Candidatus* Scalindua species are frequently detected in marine and hypersaline ecosystems and are broadly regarded as representative marine anammox bacteria [9, 12]. Notably, *Ca*. Scalindua sp. exhibit exceptional tolerance to oxygen exposure [13, 14, 15], elevated salinity [16, 17], and energy limitation [18]. Recent determination of dual isotope effects (^15^ε and ^18^ε) associated with anammox metabolism in *Ca*. Scalindua has enabled more refined estimates of its contribution to marine nitrogen loss [19, 20]. Accordingly, precise quantification of oceanic nitrogen loss requires a deeper mechanistic understanding of the oxygen tolerance and physiological responses of *Ca*. Scalindua.

Previous studies employing highly enriched *Scalindua* biomass have reported high superoxide dismutase (SOD) activity and an estimated IC_50_ of 20 µM DO, suggesting a higher oxidative stress tolerance compared to other anammox taxa [15]. However, these estimates were derived from theoretical DO concentrations based on gas–liquid equilibrium assumptions, rather than direct DO measurements in the aqueous phase. As such, the actual DO thresholds and the fundamental mechanisms underlying oxygen tolerance remain unresolved.

Further complicating this issue, reported DO concentrations associated with anammox activity in marine environments span a broad range—from 0.9 to 20 μM—likely reflecting variation in cell density, species composition, cell morphology (aggregated vs. planktonic), enrichment levels, and analytical methodologies [2, 10, 11, 14, 21]. For example, aggregated cells may exhibit apparent oxygen tolerance due to diffusion-limited oxygen penetration [22, 23], while coexisting aerobic ammonia-oxidizing bacteria (AOB) and archaea (AOA) in low-enrichment cultures may artificially reduce ambient DO [23], thereby confounding assessments of anammox oxygen tolerance. Consequently, reliance on headspace-derived DO estimates and aggregated biomass can obscure the actual oxygen concentration experienced by anammox cells, potentially leading to an overestimation of their oxygen tolerance. To address this limitation, we used a highly enriched planktonic *Ca*. Scalindua culture together with direct DO measurements, allowing a more accurate determination of oxygen tolerance and its underlying mechanisms.

Obligate anammox bacteria must employ robust protective strategies to counteract the deleterious effects of molecular oxygen and its associated reactive oxygen species (ROS). Although the molecular mechanisms underlying oxygen detoxification in anammox bacteria remain poorly characterized, genomic analyses of *Ca*. Scalindua have revealed the presence of antioxidant defense systems, including genes encoding superoxide dismutase (*sod*), catalase (*kat*), and various peroxidases [15, 24, 25, 26]—but notably not bilirubin oxidase (*bod*) [24]. Intriguingly, the gene encoding cbb₃-type cytochrome *c* oxidase (CcO), a terminal oxidase typically associated with microaerobic respiration, has also been identified in *Scalindua* sp. [12, 15, 24, 27, 28, 29]. This enzyme is hypothesized to catalyze the direct four-electron reduction of molecular oxygen to water. This reaction may be coupled to transmembrane proton translocation, thereby generating a proton motive force (PMF) that supports ATP synthesis and potentially enhances cellular survival under microoxic conditions. Despite its putative physiological relevance, transcriptional and translational analyses of cbb₃-type CcO in anammox bacteria remain limited. To date, only one study has reported homologous expression of the CcO-encoding gene and protein within a microbial consortium containing anammox bacteria [29]. However, anammox bacteria constituted only ~10% of the community, and the observed expression profiles were likely confounded by coexisting microorganisms, obscuring anammox-specific regulatory responses. Moreover, the functional role of cbb₃-type CcO, whether in oxygen detoxification or energy conservation under microoxic conditions, remains unresolved.

In this study, we quantitatively investigated the oxygen tolerance mechanisms of the marine anammox bacterium *Ca*. Scalindua sp. using a highly enriched planktonic culture system coupled with direct microoxic DO measurements. We further characterized the transcriptional dynamics of key genes involved in oxidative-stress defense and oxygen detoxification, together with CcO activity. By integrating physiological and molecular perspectives, our results refine current understanding of anammox function under fluctuating redox regimes and suggest that *Scalindua* could sustain fixed-nitrogen removal within microoxic niches of OMZs. To avoid ambiguity, we distinguish between “oxygen tolerance,” defined as the ability of *Scalindua* cells to endure oxygen exposure, and “oxygen scavenging,” referring to the enzymatic removal of oxygen.

## Materials and methods

### Enrichment culture of *Ca*. Scalindua sp.

Following previously established protocols [30, 31], *Ca*. Scalindua sp. was continuously cultivated under anoxic conditions using a membrane bioreactor (MBR) system designed to maintain dispersed, planktonic cells. The reactor volume (2 l) was kept constant via a water-level sensor, and the hydraulic retention time was set to 1 day. To ensure anoxic conditions, a continuous flow of Ar-CO₂ gas mixture (95:5, v/v) was supplied to the reactor headspace. Cultivation was conducted at 25°C using an inorganic nutrient medium containing the following components (mg l^−1^): KHCO₃ (500), KH₂PO₄ (32.9), MgSO₄·7H₂O (99), CaCl₂·2H₂O (180), FeSO₄·7H₂O (9.14), CoCl₂·6H₂O (0.24), ZnSO₄·7H₂O (0.43), MnCl₂·4H₂O (0.99), CuSO₄·5H₂O (0.25), NaMoO₄·7H₂O (0.22), NiCl₂·6H₂O (0.19), H₃BO₄ (0.007), and EDTA·2Na (21.0). Ammonium and nitrite were added as (NH₄)₂SO₄ and NaNO₂, respectively, at concentrations ranging from 2 to 12 mM depending on biomass levels (~0.2 mg-protein ml^−1^). KHCO₃ solution was degassed with high-purity N₂ gas (>99.995%) for at least 1 h prior to use. Salinity was adjusted to 3% (w/w) using artificial sea salt (SEALIFE, Marine Tech) to match the optimal conditions for *Scalindua* growth.

The metagenome-assembled genome (MAG) obtained from the enriched *Ca*. Scalindua sp. population showed 99.99% average nucleotide identity to *Ca*. Scalindua sp. SCAELEC01 (GenBank assembly accession: GCA_004282745.1) [26], indicating that the microorganism belongs to the species-level lineage *Candidatus* Scalindua sp. SCAELEC01 (NCBI Taxonomy ID: 2512262). *Scalindua* sp. shared 91.9% 16S rRNA gene sequence identity with its closest known relative, *Scalindua rubra* A (Fig. S1).

### Percoll density gradient separation

To further enhance biomass purity, Percoll-density gradient centrifugation was conducted according to previously established protocols [32]. To prepare the gradient, 6 ml of Percoll (Cytiva) was mixed with 3 ml of inorganic medium lacking ammonium and nitrite in a 15 ml centrifuge tube, and centrifuged at 10 000 × *g* for 30 min to establish a stable gradient. Biomass of *Scalindua* sp. was harvested from the MBR and pelleted by centrifugation at 14 000 × *g* for 8 min. The supernatant was discarded, and the pellet was resuspended in 1 ml of inorganic nutrient medium. The suspension was carefully layered onto the preformed Percoll gradient and centrifuged at 6000 × *g* for 30 min. The reddish anammox-enriched layer was collected using a pipette. To remove residual Percoll and nitrogen compounds, the collected biomass was washed three times by resuspension in inorganic medium followed by centrifugation at 10 000 × *g* for 8 min. The resulting purified biomass was used for subsequent oxygen inhibition experiments.

All steps of the Percoll density-gradient centrifugation procedure, except for the centrifugation itself, were performed inside an anaerobic chamber. The Percoll separation procedure was confirmed not to unduly influence anammox activity (Fig. S2).

### Anoxic batch cultivation of *Ca*. Scalindua sp. biomass

Highly purified biomass of *Ca*. Scalindua sp., obtained via Percoll density gradient centrifugation, was transferred into 50 ml of the medium in 125 ml sterile serum vials (headspace volume: 75 ml; cross-sectional area: 12.6 cm^2^) inside an anaerobic chamber (COY Laboratory Products, Model-AS). Each vial was sealed with a butyl rubber stopper and aluminum crimp cap. The headspace gas was replaced with high-purity helium (>99.9%) using a gas exchange apparatus (IP-8 model, Sanshin Industrial Co., Ltd.), and the pressure was adjusted to 1.5 atm to ensure strict anoxic conditions. Penicillin G (benzylpenicillin; 500 mg l^−1^) and allylthiourea (ATU; 100 mg l^−1^) were added to inhibit coexisting heterotrophic bacteria and aerobic AOB, respectively. The treated biomass was subsequently used for oxygen inhibition assays.

### Oxygen inhibition assay of *Ca*. Scalindua sp.

To avoid interference from atmospheric nitrogen (^28^N₂) during GC/MS analysis, pure oxygen gas (>99.9%, 1 atm) was used instead of air for all oxygen exposure experiments. Varying volumes of pure oxygen gas was injected into the headspace with a gas tight syringe before and after the addition of NH_4_^+^ and NO_2_^−^ (final concentration of 5 mM each). Thereafter, the vials were incubated at room temperature (ca. 25°C). During the batch cultivation period, the culture medium in the vial was gently stirred using a magnetic stirrer at ~200 rpm. Anammox activity was monitored by measuring the production of hybrid dinitrogen (^14 + 15^N₂), which is specifically generated via the anammox reaction between ^15^NH₄^+^ and ^14^NO₂^−^. To infer the reaction pathway, isotopic labeling was selectively applied: when both NH_4_^+^ and NO_2_^−^ were added, only NH₄^+^ was labeled with ^15^N; when NO_2_^−^ was added alone, NO₂^−^ was labeled with ^15^N.

### Specific oxygen reduction rate

Batch cultivation experiments were conducted in sealed vials (75 ml gas phase, 50 ml liquid phase) with pure oxygen injection. Specific oxygen reduction rates (SORRs) (nmol-O₂ min^−1^ mg-protein^−1^) were determined as the sum of DO depletion rate in the liquid phase and O₂ depletion rate in the headspace, normalized to biomass protein content. Measurements were performed under three substrate regimes: NH₄^+^ + NO₂^−^, NO₂^−^ alone, and NH₄^+^ alone, in the presence or absence of penicillin G (500 mg l^−1^; general bacterial inhibitor) and ATU (100 mg l^−1^; specific inhibitor of ammonia monooxygenase, AMO).

Additional assays were conducted with NH₄^+^ + NO₂^−^ in the presence of established anammox inhibitors: 21% O₂, acetylene (70 μM; Hzs inhibitor blocking the hydrazine-formation step) [33], methanol (5 and 10 mM; HDH inhibitor blocking the hydrazine-oxidation step) [34], ClO₃^−^ [0.5 and 5 mM; nitrite oxidoreductase (NXR) inhibitor] [35], PTIO [2-Phenyl-4,4,5,5-tetramethylimidazoline-1-oxyl-3-oxide; nitric oxide (NO) scavenger, 100 mg l^−1^ ≈ 0.483 mM] [36], and sodium azide (NaN₃; CcO inhibitor, 0.77 mM) [37].

### Chemical analyses

During the oxygen inhibition batch experiments, DO concentrations were continuously monitored using a non-invasive optical DO sensor (Fibox 3, PreSens Precision Sensing GmbH, Germany) [38]. Specifically, PSt3 sensor patches were affixed to the inner wall of the vials using adhesive, and measurements were conducted in the dark. Calibration was performed using a 50 g l^−1^ sodium sulfite (Na₂SO₃) solution and air-saturated water. Although fluorescence-based DO sensors have been reported to be affected by certain culture supernatants [39], no interference was observed with *Scalindua*’s cultures (Fig. S3).

Ammonium concentrations were determined using the ortho-phthalaldehyde method [40], nitrite by the naphthylethylenediamine method [41], nitrate by ion chromatography (NI-424 column, Shodex; column oven temperature: 40°C), and protein concentrations by the DC Protein Assay (Bio-Rad Laboratories) [42]. NO was determined through PTIO scavenging, and PTIO concentrations were determined by UV–Vis spectrophotometry at the absorption maximum (λ_max_ ≈ 320 nm), using calibration curves prepared with freshly dissolved standards (0–100 μM) in the same buffer system [43].

Gas samples were analysed for molecular oxygen (O_2_) and ^29^N_2_ using gas chromatography–mass spectrometry (GC–MS). The analyses were performed on a GCMS-QP2010 (Shimadzu) equipped with a CP-PoraBOND Q fused silica capillary column (25 m × 0.32 mm × 5 μm). Measurements were conducted in scan mode to detect target gases with high sensitivity and resolution. The measured headspace oxygen concentrations (O₂, %) were corrected for a systematic error arising from atmospheric oxygen contamination introduced during GC–MS sample injection. Details of the correction procedure are provided in the Supplemental Information (Fig. S4).

### Microbial community analysis

Microbial community analysis was performed on *Scalindua*-enriched biomass from two membrane bioreactors. DNA was extracted, and amplicon libraries targeting the V4 region of the 16S rRNA gene were prepared using a two-step PCR protocol. Sequencing was conducted on the Illumina MiSeq platform (2 × 300 bp), and raw reads were processed with QIIME2 (ver. 2023.7) using DADA2 for denoising and chimera removal. Taxonomic classification was carried out with the Greengenes database (ver. 13.8, 97% OTU reference). Detailed protocols are provided in the Supplemental Information.

### Transcriptional dynamics of oxygen-detoxification genes

The expression dynamics of putative oxygen detoxification genes in Ca. Scalindua sp. exposed to oxygen for 1 h and 24 h were analysed using RT-qPCR. Target genes included flavodiiron proteins (*fdp1*–*4*, *fdp*-*rd*), catalase (*kat*), rubrerythrins (*rbr1*, *rbr2*), neelaredoxin/superoxide reductase (*nlr*/*sor*), superoxide dismutase (*sod*), glutathione peroxidases (*gpx1*, *gpx2*), cytochrome *c* peroxidase (*ccp*), and cbb₃-type CcO subunits (*ccoP*, *ccoO*, *ccoN*). Gene-specific primers were designed for each target (Table S1), and transcriptional levels were normalized against hydrazine dehydrogenase (*hdh*), a stable functional marker of anammox under anoxic conditions [44]. Detailed protocols for RNA extraction, cDNA synthesis, PCR validation, and qPCR conditions are provided in the Supplemental Information.

### Cytochrome *c* oxidase activity assay

Biomass samples were collected at four stages of the oxygen-exposure batch experiment: before oxygen addition (anoxic), after oxygen addition but before substrate addition, during the decrease in DO, and after DO stabilized at a low level. Cells were harvested by centrifugation (10 000 × g, 10 min, 4°C), resuspended in Tris–HCl buffer (pH 7.2) containing 0.1 mM PMSF, and disrupted three times using a French press (120 MPa). The lysate was clarified by centrifugation (4000 × g, 5 min, 4°C), and the supernatant was ultracentrifuged (200 000 × g, 1 h, 4°C) to obtain soluble and membrane fractions. The membrane pellet was resuspended in enzyme buffer (10 mM Tris–HCl, pH 7.0; 250 mM sucrose; 1 mM DDM) and incubated for 60 min at 4°C to extract membrane proteins.

CcO activity was measured following the CYTOCOX1 kit protocol (Sigma–Aldrich). Reduced cytochrome *c* (0.22 mM) was prepared by treating oxidized cytochrome *c* with 0.5 mM Dithiothreitol for 15 min. The absorbance of reduced cytochrome *c* was adjusted to A550 = 0.2–0.3, and complete reduction was confirmed by an A550/A565 ratio of 10–20. The reaction was initiated by adding 100 μL of membrane or soluble extract, and the decrease in A550 was recorded. CcO activity, defined as the oxidation rate of reduced cytochrome *c*, was calculated using a molar extinction coefficient of 21.84 mM^−1^ cm^−1^ together with the corresponding protein concentration, and expressed as nmol min^−1^ mg-protein^−1^.

### Statistical analysis

All statistical analyses were conducted using R (version 4.5.2). SORRs and CcO activities were compared among various treatments using a one-way analysis of variance (ANOVA). When the ANOVA indicated a significant treatment effect, post hoc pairwise comparisons were performed using Tukey’s honestly significant difference (HSD) test. Statistical significance was defined as *P* < .05.

## Results

### Influence of substrate availability on oxygen reduction

Oxygen inhibition assays were performed to examine the effect of substrate availability on the oxygen reduction rate (ORR) of *Ca*. Scalindua sp. Oxygen was injected either before or after the addition of anammox substrates (NH₄^+^ and NO₂^−^).

### O_2_ injection in the presence of substrates

**Figure 1:**
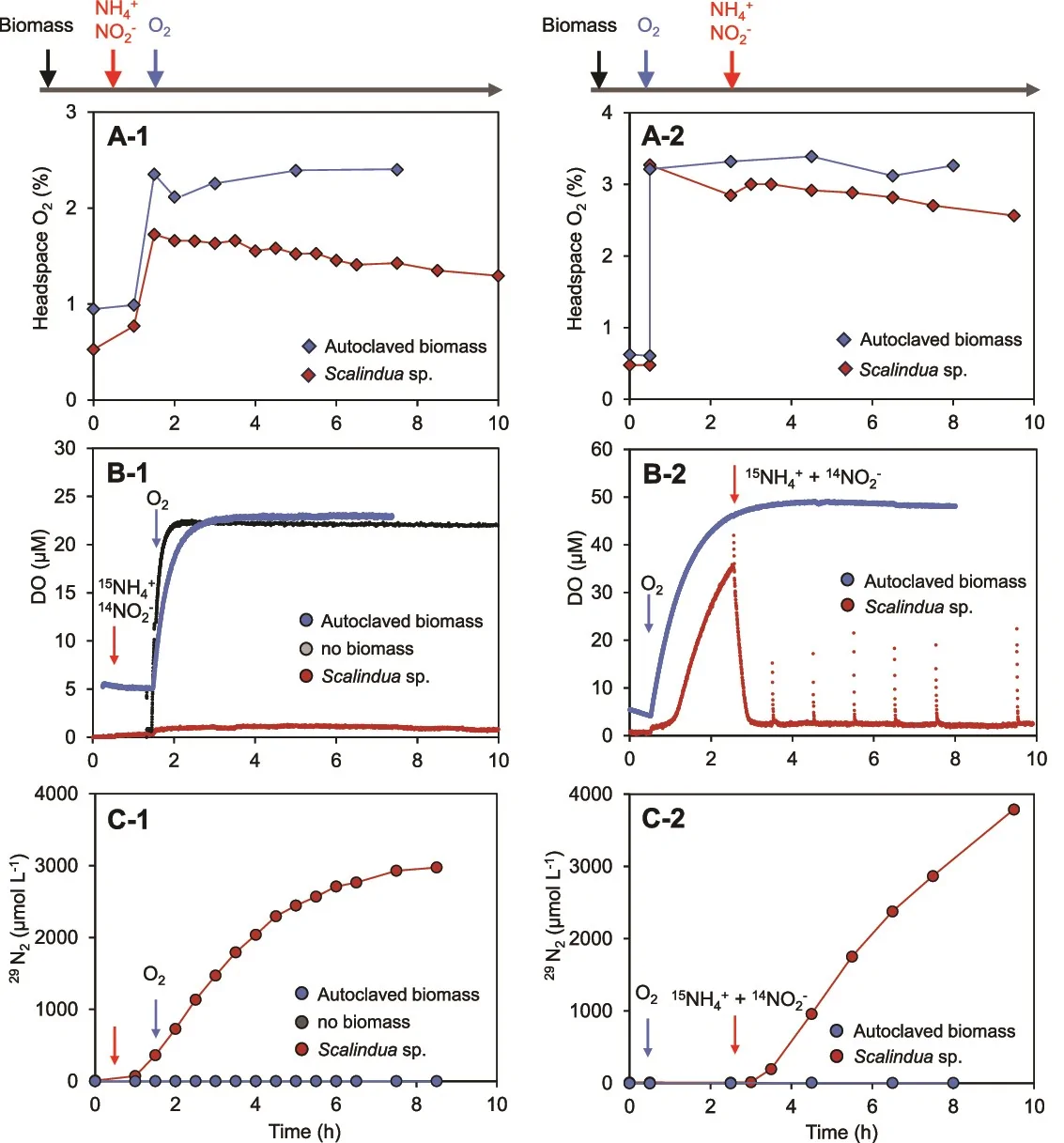
Effects of oxygen injection on DO concentration and anammox activity (^29^N₂ production) in the presence (A-1, B-1, C-1) and absence (A-2, B-2, C-2) of substrates (^15^NH₄^+^ and ^14^NO₂^−^). Pure oxygen (>99.9% O₂, 1 atm) was injected into sealed vials containing culture medium with or without anammox substrates (^15^NH₄^+^ and ^14^NO₂^−^). Time courses of headspace O₂ (%) (A), DO (B), and ^29^N₂ production (C) were monitored. In panel A, the headspace O_2_ data were not obtained for the no-biomass control. In panel C, the ^29^N₂ data points for the no-biomass control are obscured by the autoclaved-biomass data points plotted at zero. All experiments were independently performed in triplicate, yielding consistent and reproducible results. Because the sampling intervals differed among the three runs, the combined data could not be plotted with mean and standard deviation. One representative dataset is shown in Fig. 1, and the other two are provided in Supplemental information (Fig. S5).

Substrate consumption commenced immediately after the addition of ^15^NH_4_^+^ and ^14^NO_2_^−^ at 0.5 h, leading to the detection of ^29^N₂ by the first sampling point at 1.0 h (Fig. 1A–1; Fig. S5A). Pure oxygen (99.9%, 1 atm) was injected into the culture containing *Ca*. Scalindua biomass and anammox substrates at 1.5 h, and this treatment did not inhibit N₂ production (Fig. 1C–1, Fig. S5A).

DO concentrations remained stable at several μM, whereas the headspace O₂ levels decreased steadily. Control experiments conducted without biomass or with autoclaved biomass showed a rapid increase in DO concentration following O₂ injection, stabilizing at ~23 μM -close to the theoretical saturation level of 29 μM- within 1 h (Fig. 1B–1). Headspace O₂ concentrations remained unchanged, and no ^29^N₂ production was observed. These results clearly indicate that *Ca*. Scalindua biomass actively consumes DO originating from the headspace.

### O_2_ injection in the absence of substrate

**Figure 2:**
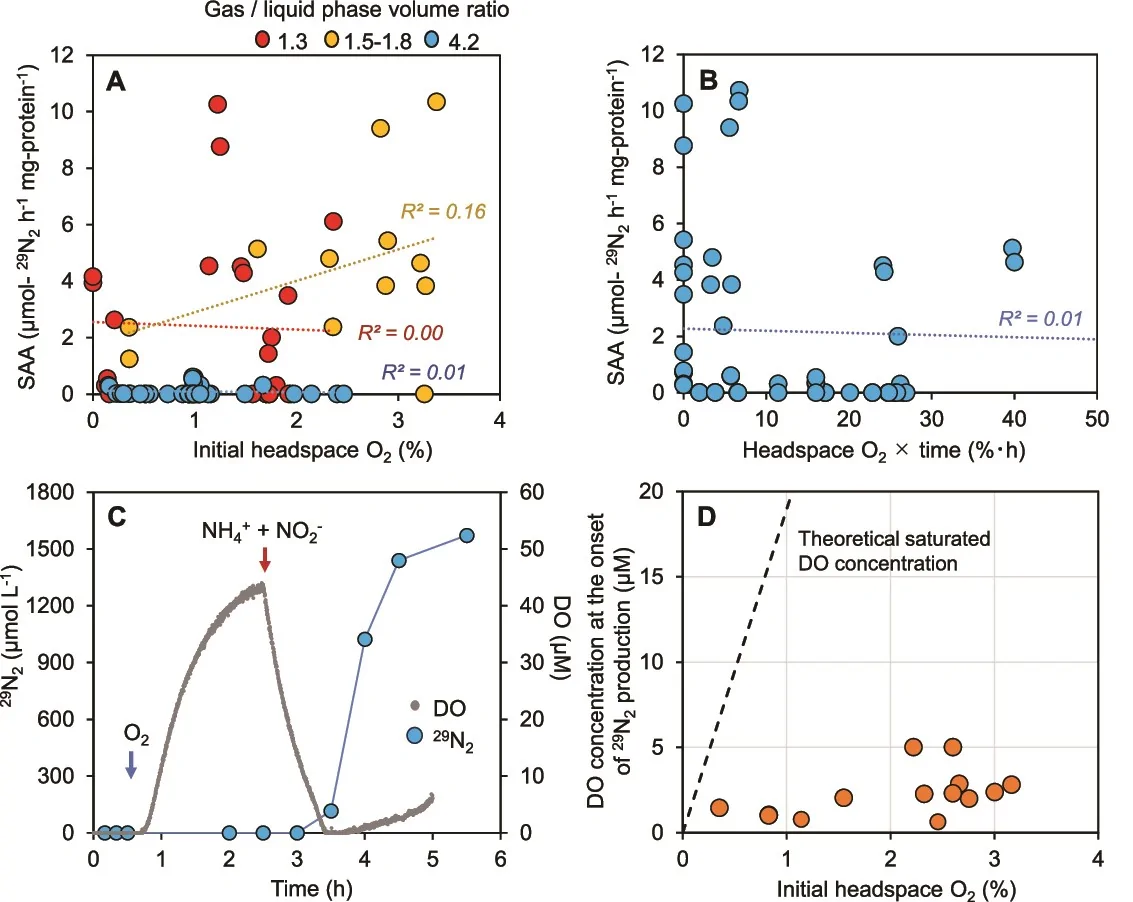
Relationship between initial headspace O₂ concentration and anammox activity. Specific anammox activity (SAA) was plotted against (A) the initial headspace O₂ concentration (%) and (B) the cumulative O₂ exposure (O₂ concentration (%) × exposure time (h)). ^29^N₂ production consistently initiated when DO declined below ~5 μM, regardless of the initial headspace O₂ concentration (C). The DO threshold for the onset of ^29^N₂ production remained invariant across treatments with differing initial headspace O₂ levels (D). The dotted line represents the theoretical DO saturation, calculated from the corresponding initial headspace O₂ concentration.

In a contrasting experimental setup, oxygen was injected at 0.5 h into an inorganic nutrient medium containing only *Ca*. Scalindua biomass, followed by the addition of NH_4_^+^ and NO_2_^−^ at 2.5 h (Fig. 1A–2). Following O₂ injection, DO concentrations increased rapidly, then declined sharply upon the addition of ^15^NH₄^+^ and ^14^NO₂^−^ (Fig. 1B–2). After DO stabilized at low micromolar levels, a gradual decrease in headspace O₂ concentration (%) was observed, indicating sustained oxygen consumption. Notably, despite relatively high headspace O₂ levels (~ 3%), ^29^N₂ production commenced when DO concentrations fell below ~5 μM, suggesting that anammox activity resumed under microoxic conditions (Fig. S5B). Under the lower biomass condition, both DO and headspace O₂ concentrations declined more gradually than in the higher biomass setup (data not shown). Control experiments with autoclaved biomass exhibited a rapid increase in DO following O₂ injection, which subsequently stabilized. No change in headspace O₂ was observed after the addition of ^15^NH₄^+^ and ^14^NO₂^−^, and no ^29^N₂ production was detected (Fig. 1A–2, B-2, C-2). These findings clearly demonstrate that oxygen consumption was biologically mediated.

### Factors controlling anammox activity

No correlation was observed between the initial headspace O₂ concentration (%) and specific anammox activity (SAA) (Fig. 2A). Likewise, SAA showed no significant correlation with oxygen exposure intensity, defined as the product of initial headspace O₂ or DO concentration and exposure time (Fig. 2B). In these batch incubations, SAA was assessed based on ^29^N₂ production over a relatively short period (5–6 h). Notably, a higher gas-to-liquid volume ratio in the vial increases the total amounts of O₂ that can dissolve into the liquid phase, even when headspace O₂ concentration remains constant. Under such conditions, DO may not decrease sufficiently within this timeframe to permit the onset of anammox activity, resulting in undetectable SAA. However, given the reversible nature of anammox inhibition (see next section), ^29^N₂ production may resume upon prolonged incubation. In contrast, anammox activity consistently initiated when DO concentrations dropped below ~5 μM (Fig. 2C and D). These findings suggest that final DO concentration, rather than the initial headspace O₂ levels or cumulative oxygen exposure, is the critical factor governing the onset of anammox activity. Previous studies have estimated oxygen tolerance parameters such as IC_50_ and DO_max_ by plotting SAA against theoretical DO values calculated from the corresponding initial headspace O₂ concentrations [15]. This approach may overestimate the true oxygen tolerance of anammox bacteria.

### Impact of final dissolved oxygen concentration versus exposure intensity on anammox activity

**Figure 3:**
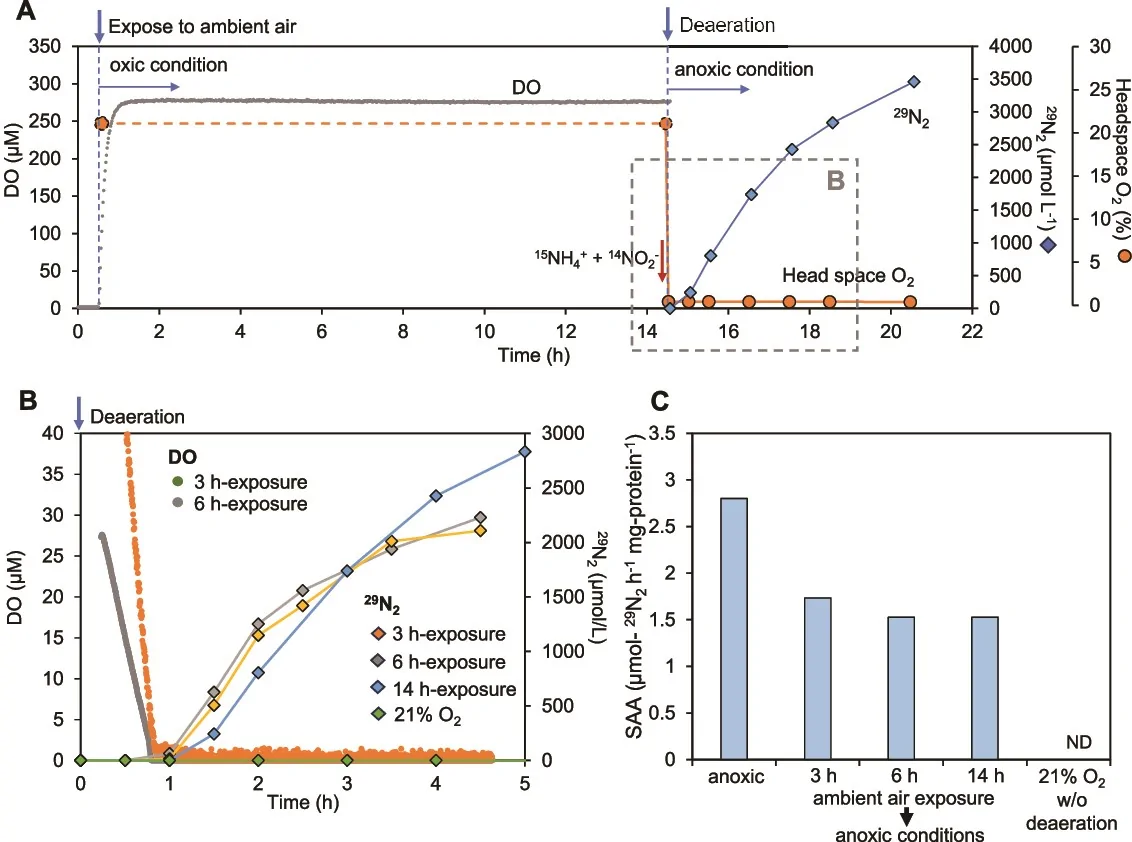
Effect of ambient O₂ exposure duration on specific anammox activity (SAA). (A) Anammox activity recovered even after 14 h of exposure to ambient air (21% O₂), as long as the DO concentration was reduced to a few μM by deaeration (as indicated by arrow). (B) Enlarged view showing the relationship between DO concentration and the onset of ^29^N₂ formation across different O₂ exposure durations. DO data were not obtained for the 14-h exposure experiment. (C) SAA following deaeration was lower than that of the strictly anoxic positive control, indicating that prior O₂ exposure may have partially and irreversibly inhibited anammox activity.

To determine whether anammox activity is governed by final DO concentration rather than oxygen exposure intensity, *Ca*. Scalindua cultures were exposed to ambient air (21% O₂) for varying durations, followed by deaeration to restore anoxic conditions (Fig. 3A). ^29^N₂ production resumed immediately after 3, 6, and 14 h of air exposure once anoxia was re-established (Fig. 3B). However, prolonged exposure led to a gradual decline in N₂ formation, with SAA values ~45% lower than those observed under strictly anoxic conditions (Fig. 3C). In contrast, cultures maintained continuously at 21% O₂ without deaeration exhibited persistently high DO concentrations (~300 μM) and no detectable anammox activity (Fig. 3C). These results collectively demonstrate that the onset of anammox activity is determined by the final DO concentration rather than the duration or cumulative intensity of oxygen exposure.

### Reversibility of oxygen inhibition and implications for reactive oxygen species detoxification in *Scalindua*

Although 14 h of exposure to ambient air (21% O₂) induces oxygen-mediated inhibition of anammox activity, this inhibition is reversible upon re-establishment of anoxic conditions. The rapid recovery suggests that *Ca*. Scalindua possesses potent enzymatic systems capable of efficiently detoxifying ROS including superoxide anion (O₂^•-^) and hydrogen peroxide (H_2_O_2_), which are otherwise highly reactive and capable of inflicting irreversible damage to DNA and cellular components.

Based on changes in headspace oxygen concentration following O₂ injection, the oxygen consumption rate was estimated to be 0.772 μmol h^−1^ (Fig. S6A-1, B-1). Concurrently, extracellular hydrogen peroxide (H₂O₂) accumulated at a rate of 0.003 μmol h^−1^ (Fig. S6C-1), accounting for ~0.4% of the total oxygen consumption. Notably, this level of H₂O₂ accumulation was comparable to that observed under without O_2_ injection (Fig. S6C-2). Although intracellular H₂O₂ concentrations are presumed to exceed extracellular levels, the overall fraction of oxygen converted to ROS remained remarkably low. These results suggest that molecular oxygen is either directly reduced to water or that ROS formed during oxygen reduction are rapidly and efficiently detoxified by antioxidant enzymes, such as SOD and various peroxidases.

Consequently, *Scalindua* cells are unlikely to suffer lethal damage to DNA or cellular components due to ROS accumulation. Instead, oxygen exposure appears to transiently inhibit specific enzymatic functions, with cellular metabolic activity recovering promptly once DO concentrations fall below a critical threshold (i.e. ~5 μM).

### Effect of different substrate additions on oxygen consumption

**Figure 4:**
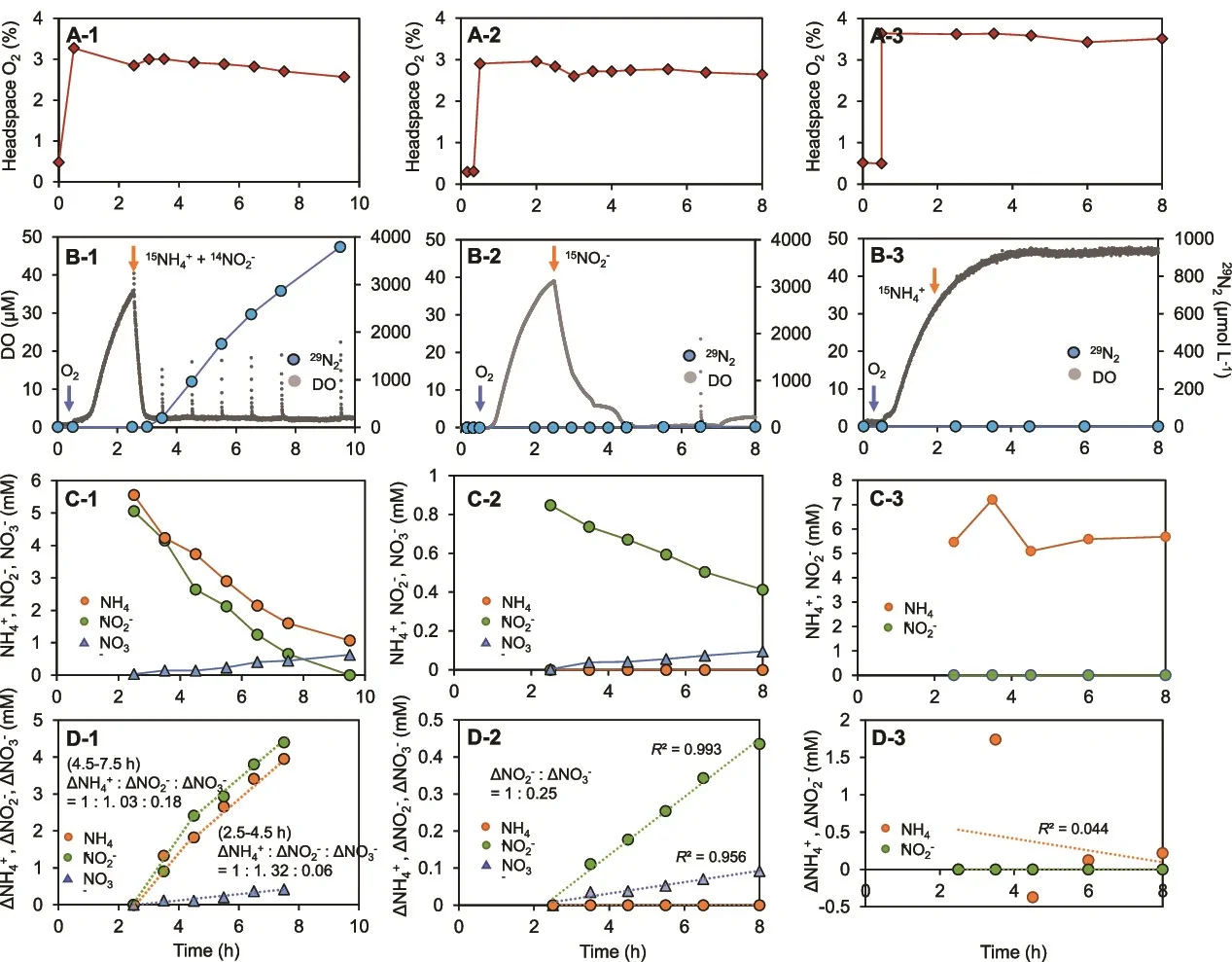
Oxygen consumption dynamics following different nitrogen substrate additions. Time-dependent changes in (A) headspace O₂ (%), (B) DO and ^29^N₂ production, (C) concentrations of NH_4_^+^, NO₂^−^ and NO₃^−^, and (D) corresponding amounts of NH₄^+^ consumed (ΔNH₄^+^), NO₂^−^ consumed (ΔNO₂^−^), and NO₃^−^ produced (ΔNO₃^−^) were monitored following the addition of various nitrogen substrates: NH₄^+^ + NO₂^−^ (−1), NO₂^−^ alone (−2), and NH₄^+^ alone (−3). In panel C-3, NO_3_^−^ was not measured. All experiments were performed in triplicate, yielding consistent and reproducible results. Because the sampling intervals differed among the three runs, the combined data could not be plotted with mean and standard deviation. One representative dataset is shown in Fig. 4, and the other two are provided in Supplemental information (Figs S7 and S8).

We monitored DO dynamics following the addition of various nitrogen substrates: combined NH₄^+^ and NO₂^−^, NO₂^−^ alone, and NH₄^+^ alone (*n* = 3 for each condition, Fig. 4). A pronounced increase in DO was observed immediately after oxygen injection, followed by a rapid decline upon the addition of NH₄^+^ and NO₂^−^. Concurrent production of ^29^N₂ confirmed the onset of anammox activity. The stoichiometric ratios of NH₄^+^ consumed to NO₂^−^ consumed and NO₃^−^ produced were ΔNH₄^+^: ΔNO₂^−^: ΔNO₃^−^ = 1: 1.32: 0.06 prior to DO depletion (2.5–4.5 h), and 1: 1.03: 0.18 following DO depletion (Fig. 4D-1). These shifts suggest that under conditions of substantial oxygen consumption, NO_2_^−^ was more extensively utilized, whereas NO_3_^−^ production remained limited. This result suggests the involvement of alternative NO_2_^−^ oxidation or reduction pathways, potentially coupled to oxygen consumption. More pronounced deviations in stoichiometry were observed in experiments conducted with lower initial concentrations of NH₄^+^ and NO₂^−^ (Fig. S7).

In the NO₂^—^only addition experiment, rapid DO consumption was observed alongside NO₂^−^ depletion (Fig. 4A-2, B-2, C-2, D-2). Approximately 0.43 mmol of NO₂^−^ was consumed, yet only 0.095 mmol of NO₃^−^ was produced, yielding a stoichiometric ratio of ΔNO₂^−^: ΔNO₃^−^ = 1: 0.25 (Fig. 4D-2). This deviates significantly from the canonical aerobic NO₂^−^ oxidation pathway mediated by NXR, which typically produces NO₃^−^ at a 1:1 ratio in the absence of intermediate products. This suggests that the observed reaction does not conform to conventional NO₂^−^ oxidation mechanisms. Reproducibility of the experiment was verified by performing three independent replicates (Fig. S8).

In contrast, the addition of NH₄^+^ alone did not result in significant DO consumption or NH₄^+^ depletion (Fig. 4A-3, B-3, C-3). As negative controls, no oxygen consumption was detected in autoclaved biomass or in the absence of biomass and substrate (Fig. S9), confirming the biological nature of the observed oxygen-consuming activity. The observed patterns strongly suggest that oxygen removal by *Scalindua* biomass is governed by a substrate-dependent regulatory mechanism.

### Confirmation of nitrite disproportionation

**Figure 5:**
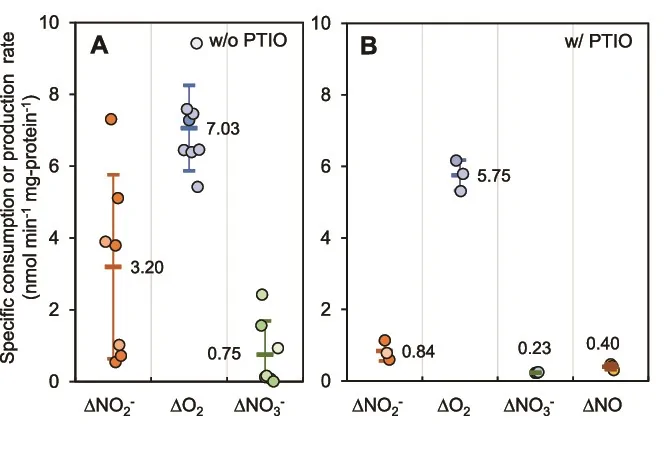
Comparative rates of NO₂^−^ depletion, oxygen consumption, NO₃^−^ production, and NO production (PTIO reduction) in batch incubations supplied only NO₂^−^ as the sole electron donor without PTIO (A) and with PTIO (B). (A) The average stoichiometric ratio of 1: 2.8: 0.3 (ΔNO₂^−^: ΔO₂: ΔNO₃^−^) deviates strongly from the canonical 1: 0.5: 1 expected for aerobic nitrite oxidation, indicating that canonical aerobic NO₂^−^ oxidation did not occur. (B) The ratio of 1: 6.8: 0.28: 0.48 (ΔNO₂^−^: ΔO₂: ΔNO₃^−^: ΔNO(PTIO)) is consistent with NO₂^−^ disproportionation to NO₃^−^ and NO (3 NO₂^−^ + 2 H^+^ → 2 NO + NO₃^−^ + H₂O), but the extent of O₂ consumption exceeds that explainable by NO-dependent pathways, implying additional O₂-consuming processes.

In the NO₂^–^only addition experiment, oxygen consumption cannot be explained by conventional aerobic NO₂^−^ oxidation. To confirm the possible occurrence of nitrite disproportionation reaction, the NO₂^–^only addition experiments were conducted with the NO-scavenging PTIO. PTIO-mediated NO scavenging resulted in an ~18% reduction in the oxygen consumption rate compared with untreated cultures (Fig. 5). Concurrently, NO₂^−^ depletion was accompanied by PTIO consumption (i.e. NO production) and NO₃^−^ formation, resulting in a stoichiometric ratio of NO₂^−^: NO (PTIO): NO₃^−^ = 1: 0.48: 0.28. These findings suggest that, under conditions where only NO₂^−^ is supplied in the absence of NH₄^+^, *Scalindua* sp. may carry out NO₂^−^ disproportionation to NO and NO₃^−^ (3NO₂^−^ + 2H^+^ → 2NO + NO₃^−^ + H₂O) via a pathway that appears to operate independently of the canonical anammox reaction.

### Microbial community analysis of enriched *Scalindua* biomass

FISH analysis using the Scal1129b probe revealed that *Scalindua* species accounted for over 99% of total bacterial cells, indicating minimum presence of coexisting bacteria (Fig. S10A). Amplicon sequence variants (ASVs) with a relative abundance ≥0.5% were included in the analysis. A total of two ASVs were identified in biomass 1 and 3 in biomass 2 (Fig. S10B), indicating that the enriched *Scalindua* cultures exhibited high purity with minimal coexisting microbial diversity. Taxonomic classification at the order level revealed a clear dominance of *Ca*. Brocadiales (genus *Scalindua*), comprising 98.2% of biomass 1 and 94.6% of biomass 2 (Fig. S10B). Notably, NGS, FISH, and qPCR analyses confirmed that no anammox taxa other than *Scalindua* sp. were detected in either biomass sample. The second most abundant order was *Rhizobiales*, accounting for 0.9% in biomass 1 and 2.4% in biomass 2. *Rhodobacterales* was detected exclusively in biomass 2 at 1.3%, while other minor populations, such as *Rhodospirillales*, represented less than 0.5%. Furthermore, aerobic AOB, AOA, and nitrite-oxidizing bacteria (NOB) were also absent in both samples.

### Transcriptional responses of key oxygen-detoxification genes following oxygen exposure

Distinct electrophoretic bands of amplified cDNA were observed at all time points, before oxygen exposure, and 1 h and 1 day after exposure, while no bands appeared in non-reverse-transcribed controls (Fig. S11). This confirms the absence of genomic DNA contamination and supports the validity of the RT-qPCR results.

**Figure 6:**
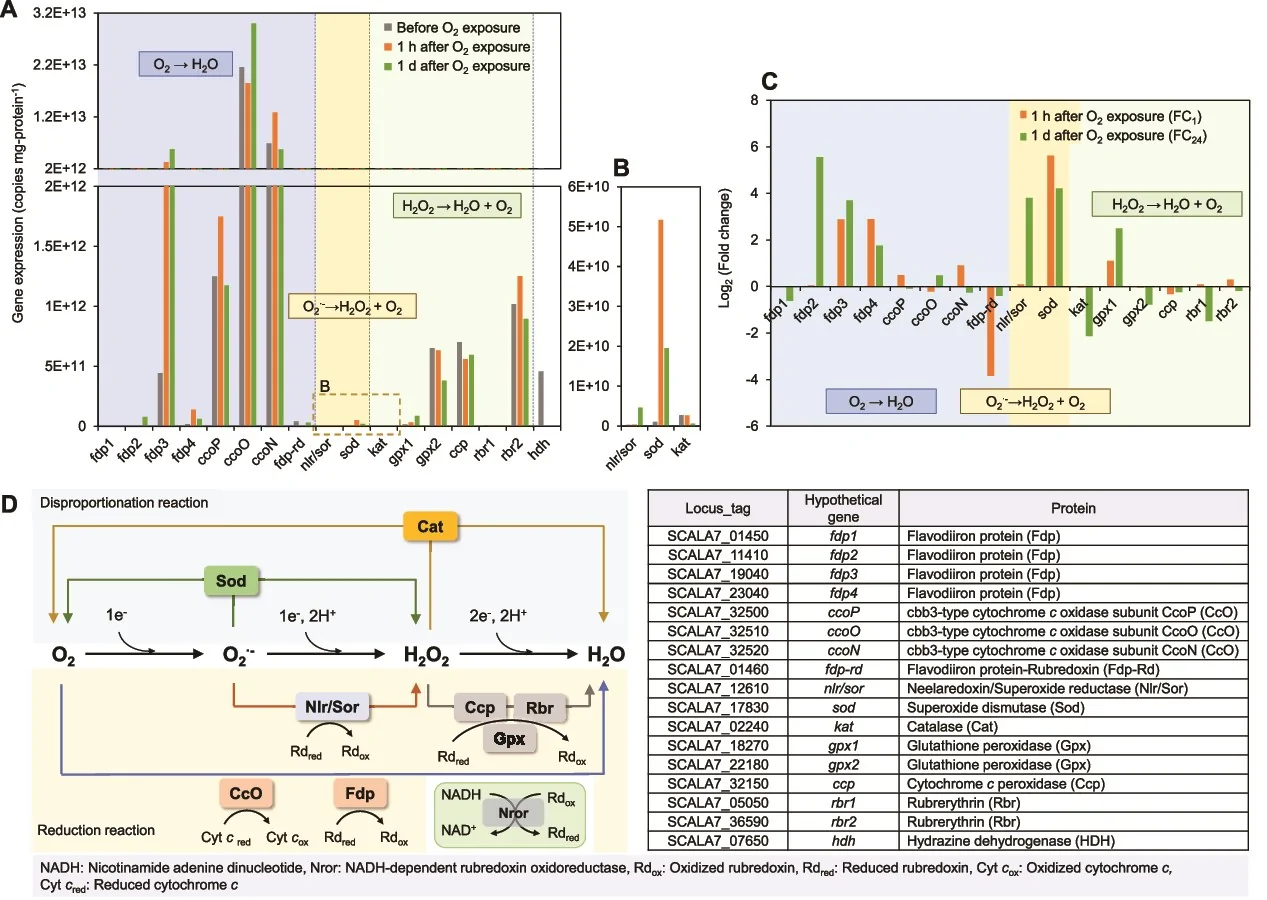
Effect of oxygen exposure on the expression of oxygen detoxification-related genes in *Scalindua* sp. (A) Gene expression levels of putative oxygen detoxification-related genes and (B) enlarged view showing the gene expression levels of neelaredoxin/superoxide reductase (*nlr*/*sor*), superoxide dismutase (*sod*), and catalase (*kat*). (C) Log₂ fold changes were calculated based on gene expression in *Scalindua* biomass before O_2_ exposure, and at 1 h (Log₂ (FC₁)) and 24 h (Log₂ (FC₂₄)) post-exposure. Genes with |Log₂ (fold change)| > 1.0 were defined as differentially expressed. (D) A proposed dual-layered reduction mechanism of molecular oxygen and ROS in *Scalindua* sp. suggests a hierarchical strategy for oxidative stress mitigation, wherein primary oxygen reduction to water is mediated by high-affinity terminal oxidases (CcO) and Fdps, while secondary ROS detoxification involves dedicated enzymes such as superoxide dismutase (Sod), superoxide reductase (Nlr/Sor), and peroxidases (i.e. Ccp, Rbr and Gpx).

The expression level of hydrazine dehydrogenase (*hdh*, SCALA7_07650), used as a reference gene for comparison, was 4.6 × 10^11^ copies mg-protein^−1^ (Fig. 6A). Superoxide dismutase (*sod*, SCALA7_17830) showed an expression level of 10^10^ copies mg-protein^−1^ (Fig. 6B). In contrast, the cbb₃-type CcO subunits (*ccoP*, *ccoO*, and *ccoN,* SCALA7_32500, SCALA7_32510, and SCALA7_32520, respectively) exhibited highest expression levels of 10^12^–10^13^ copies mg-protein^−1^. A-type flavodiiron protein (*fdp3*, SCALA7_19040) was expressed at 2 × 10^11^ copies mg-protein^−1^, glutathione peroxidase (*gpx2*, SCALA7_22180) at 2–3 × 10^11^ copies mg-protein^−1^, cytochrome *c* peroxidase (*ccp*, SCALA7_32150) at 2–4 × 10^11^ copies mg-protein^−1^, and rubrerythrins (*rbr2*, SCALA7_36590) at 4–6 × 10^11^ copies mg-protein^−1^. These expression levels were comparable to or higher than those of *sod* and *hdh*.

The expression levels of *ccoP*, *ccoO*, and *ccoN* showed only modest changes following oxygen exposure (Log₂ (FC₁) < 1.0) (Fig. 6C), suggesting that its basal level is sufficient to cope with oxygen toxicity. In contrast, superoxide dismutase (*sod*) exhibited a substantial increase (Log₂ (FC₁) and Log₂ (FC₂₄) > 4.2), as did neelaredoxin/superoxide reductase (*nlr*/*sor*, SCALA7_12610) (Log₂ (FC₂₄) ≈ 4). Furthermore, A-type flavodiiron proteins (*fdp2,* SCALA7_11410, *fdp3,* SCALA7_19040, and *fdp4*, SCALA7_23040) were markedly upregulated (Log₂ (FC) > 2.5), indicating a pronounced transcriptional response to oxygen stress.

### Response of cbb₃-type cytochrome *c* oxidase to O_2_ exposure

**Figure 7:**
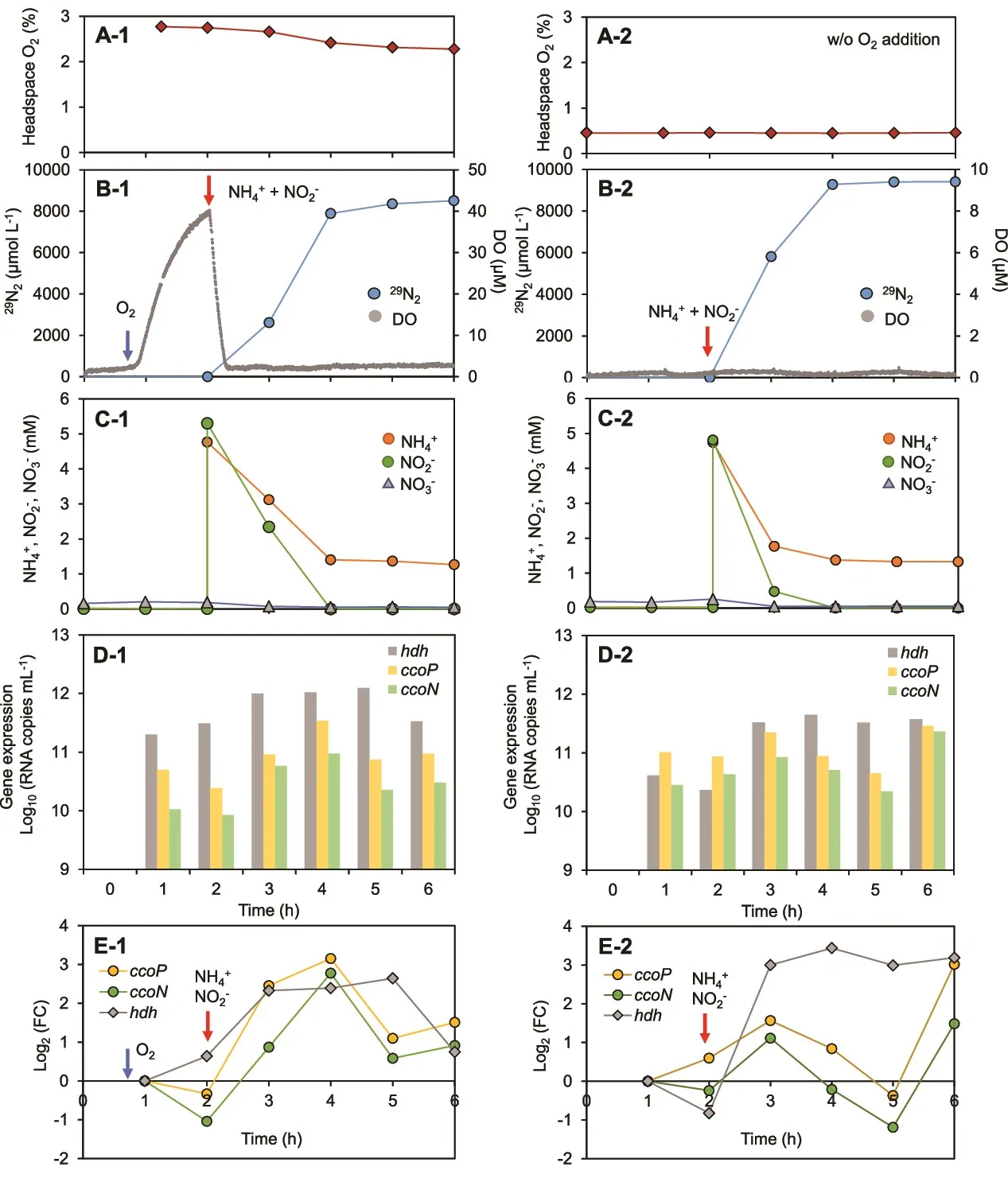
Transcriptional response of cbb₃-type cytochrome *c* oxidase subunit genes (*ccoP* and *ccoN*) to oxygen exposure. Time-dependent changes in (A) headspace O₂ concentration (%), (B) DO and ^29^N₂ production, (C) concentrations of NH_4_^+^, NO₂^−^ and NO₃^−^, (D) expression levels of *ccoP and ccoN* genes with *hdh* gene as a marker of anammox activity, and (E) corresponding Log₂ fold changes following oxygen exposure were simultaneously quantified. Measurements were conducted under two conditions: with oxygen addition (−1) and without oxygen addition (−2).

To rigorously evaluate the transcriptional response of *cco* genes to oxygen exposure, we quantified *cco* gene expression dynamics in relation to concurrently measured DO, ^29^N₂ production, and substrate concentrations (Fig. 7). Upon oxygen addition, DO levels increased sharply, followed by a rapid decline upon the addition of NH₄^+^ and NO₂^−^ 2 h later. ^29^N₂ production commenced once DO concentrations reached low micromolar levels. Expression of the *hdh* gene increased markedly in response to substrate addition and the onset of N₂ production (Log₂ (FC) = 2.0-3.0). In contrast, the transcript levels of *ccoP* and *ccoN* decreased during the initial rise in DO, but subsequently increased as DO declined following substrate addition, reaching Log₂ (FC) ≈ 3 at 2 h after the addition, and then shifted again toward a decreasing trend. Notably, similar transcriptional dynamics were observed even under oxygen-free conditions, where DO remained near the detection limit, suggesting that *cco* gene expression is regulated not by oxygen availability but rather by the presence of substrate.

### Specific oxygen consumption rates under various substrate and inhibitor conditions

**Figure 8:**
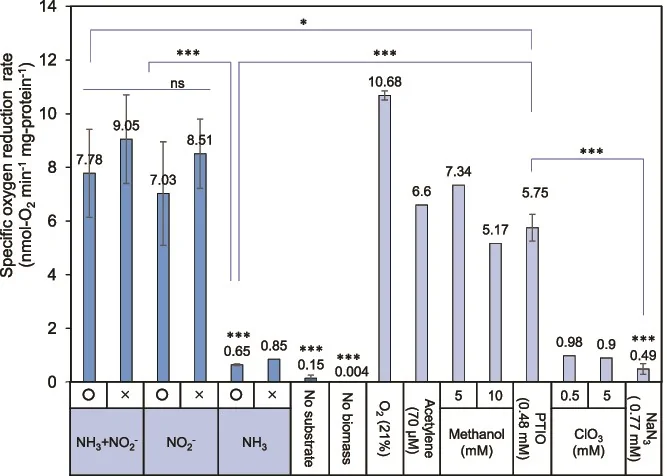
Specific oxygen consumption rates (SORR, nmol-O₂ min^−1^ mg-protein^−1^) under various substrate and inhibitor conditions. SORRs were measured under three substrate conditions: NH₄^+^ + NO₂^−^, NO₂^−^ alone, and NH₄^+^ alone in the presence (○) or absence (×) of penicillin G (a general bacterial inhibitor) and allylthiourea (ATU; a specific inhibitor of AMO). Additional measurements were performed using NH₄^+^ + NO₂^−^ as substrates with anammox-specific inhibitors: 21% O₂, acetylene, methanol, PTIO (2-Phenyl-4,4,5,5-tetramethylimidazoline-1-oxyl-3-oxide; a NO scavenger), ClO₃^−^ (an NXR inhibitor), and sodium azide (NaN₃; a potent CcO inhibitor). For each experimental condition, the independent batch incubations were performed at least three times (*n* ≥ 3), and the mean values with standard deviations are shown. Statistical comparisons were performed only for conditions with at least three independent biological replicates. Due to limited biomass availability, the acetylene-, methanol-, and ClO₃^−^ addition experiments were conducted only once and were therefore evaluated qualitatively without statistical testing. For datasets with sufficient replication, statistical significance was determined using one-way ANOVA followed by Tukey’s HSD test, with significant differences indicated by asterisks (^*^P < .05, ^**^P < .01, ^***^P < .001). ns: not significant.

Under the NH_4_^+^ + NO₂^−^ substrate condition, the SORR was 7.78 ± 1.64 nmol-O_2_ min^−1^ mg-protein^−1^ in the presence of penicillin G and ATU, and 9.05 ± 1.65 nmol-O_2_ min^−1^ mg-protein^−1^ without inhibitors, suggesting that a minor fraction of oxygen consumption may be attributable to coexisting bacteria (Fig. 8). A similar trend was observed under the NO₂^−^ only conditions, whereas SORR was negligible with NH_4_^+^ alone. The NH₄^+^-only condition exhibited significantly lower SORRs than all NH_4_^+^ + NO₂^−^ and NO₂^−^ containing treatments (*P* < .001). Oxygen consumption was essentially undetectable under substrate-free and biomass-free control conditions.

When the headspace was filled with ambient air (21% O₂), the SORR exceeded 10 nmol-O_2_ min^−1^ mg-protein^−1^, indicating that oxygen dissolution from the gas phase was rate-limiting under standard experimental conditions (headspace O₂ ≈ 3%). The behavior reflects the intrinsic oxygen-scavenging capacity of *Scalindua* sp.

In treatments with anammox-specific inhibitors [acetylene (70 μM) and methanol (5–10 mM)], ^29^N₂ production was completely suppressed. Nevertheless, the SORR remained comparable to those observed in cultures supplied with NH₄^+^ + NO₂^−^ or with NO₂^−^ alone, demonstrating that oxygen consumption was independent of core anammox metabolism. Consistently, PTIO treatment moderately inhibited the SORR, yielding a rate of 5.75 ± 0.50 nmol-O₂ min^−1^ mg-protein^−1^, which was significantly lower than the untreated NH₄^+^ + NO₂^−^ and NO_2_^−^ control (*P* < .05). These results indicate that abiotic reactions between NO and O₂ (e.g. 2NO + O₂ → 2NO₂, followed by 2NO₂ + H₂O → HNO₃ + HNO₂) occurred to some extent, yet they were not the dominant oxygen scavenging mechanism under the conditions examined.

In contrast, the addition of ClO₃^−^, a specific inhibitor of NXR, resulted in complete cessation of ^29^N₂ production and a pronounced decrease in oxygen consumption. Given that *Scalindua* sp. harbors NXR and catalyzes NO₂^−^ oxidation as part of its anammox metabolism, these results strongly suggest that NO₂^−^ oxidation serves as a key electron-donating process linked to oxygen reduction. This interpretation is further supported by the negligible SORR observed with NH_4_^+^ alone and the temporal decoupling of oxygen consumption from N₂ production.

Furthermore, the alternative electron-donating pathway—hydrazine (N₂H₄) oxidation—was likely impaired due to the absence of NO, which is typically produced via Nir-mediated NO₂^−^ reduction. Thus, in the presence of ClO₃^−^, both NO₂^−^ oxidation and N₂H₄ oxidation were suppressed, leading to a marked inhibition of oxygen reduction.

To assess the potential involvement of cbb₃-type CcO in oxygen reduction, *Scalindua* sp. cultures were treated with sodium azide (NaN₃, 0.77 mM), a specific inhibitor of CcOs. Following NaN₃ treatment, SAA remained at ~80%, whereas SORR declined sharply to 0.49 ± 0.20 nmol-O₂ min^−1^ mg-protein^−1^. NaN₃ showed the strongest inhibition, yielding significantly lower SORR values than all other treatments (*P* < .001). This functional divergence indicates that anammox activity is not directly coupled to oxygen removal and supports the involvement of cbb₃-type CcO in oxygen reduction by *Scalindua* sp.

### Activity measurements of cytochrome *c* oxidase

**Figure 9:**
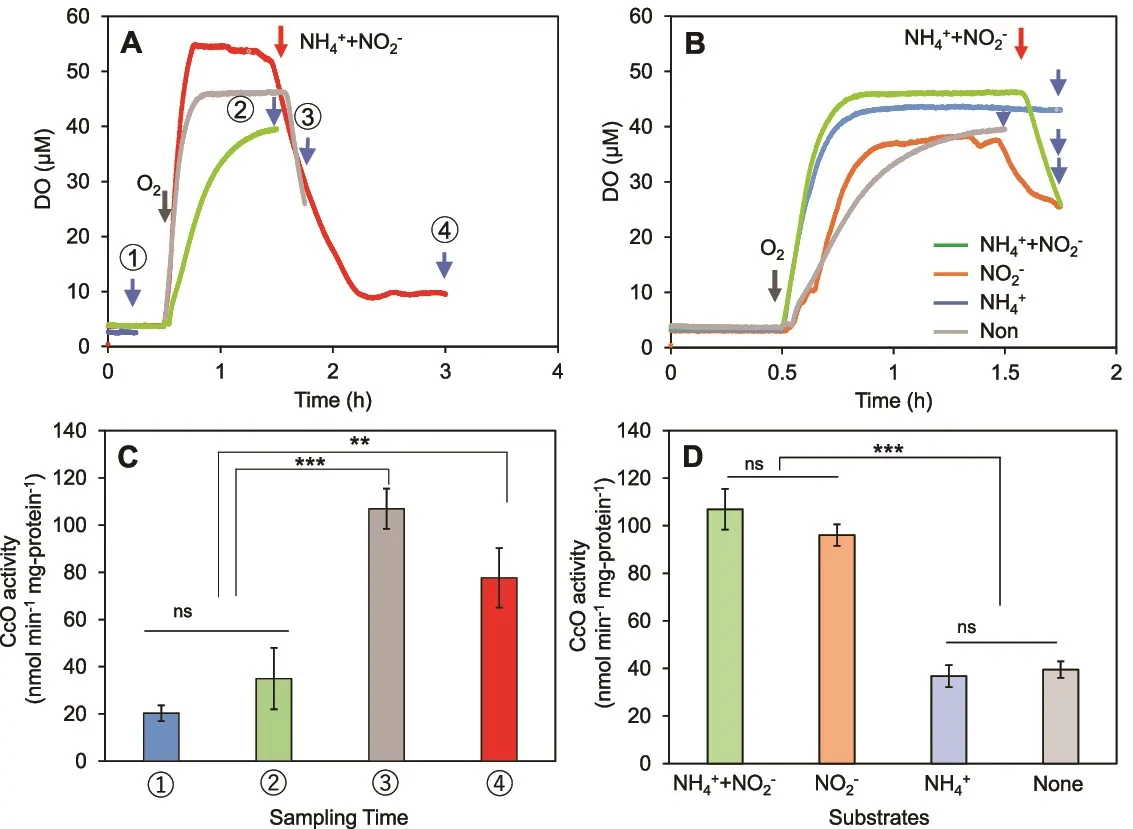
Activity measurements of cbb₃-type CcO during oxygen exposure batch experiments. Biomass samples were collected at four stages: (i) before oxygen addition (anoxic), (ii) after oxygen addition but before substrate addition, (iii) during the decrease in DO, and (iv) after DO stabilized at a low level (A and C). The effect of individual substrates (NH_4_^+^ + NO_2_^−^, NO_2_^−^ alone, NH_4_^+^ alone, and none) was then assessed 15 min after their addition (B and D). Time-course profiles of DO concentration prior to biomass sampling (A and B) and CcO activity (C and D). The blue arrows in panels A and B indicate the time points at which biomass samples were collected. For each experimental condition, three independent batch incubations were performed (*n* = 3), and the mean values with standard deviations are shown. Statistical significance was assessed by one-way ANOVA followed by Tukey’s HSD test. Significant differences are indicated by asterisks (^**^P < .01, ^***^P < .001). ns: not significant.

CcO activity was quantified at four key stages during the oxygen-exposure batch experiments: before oxygen addition, after the rise in DO but prior to substrate addition, during the subsequent DO decline following substrate addition, and after DO had decreased and stabilized at low levels. CcO activity rapidly increased from 20–35 nmol min^−1^ mg-protein^−1^ before substrate addition to 78–107 nmol min^−1^ mg-protein^−1^ after NH₄^+^ and NO₂^−^ addition, with post-addition activities significantly higher than pre-addition levels (*P* < .05) (Fig. 9A and C). No significant difference was detected between the two pre-addition samples. These results indicate that CcO activity is induced more strongly by substrate addition than by oxygen exposure.

The effect of individual substrates was then assessed 15 min after substrate addition (Fig. 9B and D). CcO activity increased significantly with the addition of NH₄^+^ and NO₂^−^ or NO₂^−^ alone (*P* < .001), whereas addition of NH₄^+^ alone produced no detectable induction, indicating that the presence of NO₂^−^ is likely the key driver of CcO activation. These responses closely paralleled transcriptional response of CcO subunit genes (Fig. 7E) and the substrate-dependent differences in SORR (Fig. 8).

## Discussion

### Specific oxygen reduction rate

ORR and SOD activity are important determinants related to the oxygen tolerance of anaerobic bacteria [15, 45, 46]. Under microoxic conditions with headspace oxygen concentration of ~3%, *Scalindua* sp. biomass exhibited a moderate oxygen-reduction rate of roughly 10 nmol-O₂ min^−1^ mg-protein^−1^ in this study. Even after 14 h of exposure to ambient air (21% O₂), anammox activity, indicated by ^29^N₂ production, commenced once the DO concentration decreased to ~5 μM (Fig. 3). In our previous study [15], *Scalindua* sp. exhibited high SOD activity (22.6 ± 1.9 U mg-protein^−1^; where 1 U corresponds to the degradation of 1 μmol of superoxide radicals (O₂^•-^) per minute), a value roughly 1000-fold higher than the SORR (~10 nmol-O₂ min^−1^ mg-protein^−1^) and 100-fold higher than the CcO activity (~100 nmol min^−1^ mg-protein^−1^) measured in the present study. This exceptionally high ROS detoxification capacity enables the rapid and complete elimination of ROS generated during oxygen reduction (Fig. S6C), thereby preventing fatal damage to DNA and other cellular components. Such a protective mechanism provides a physiological basis for the reversible oxygen tolerance observed in *Scalindua* sp.

A previous study demonstrated that oxygen-tolerant anaerobes, such as *Clostridium perfringens* and *Bifidobacterium*—capable of surviving 48 to over 72 h in the presence of oxygen—exhibited SORR ranging from 0.8 to 2.5 nmol-O_2_ min^−1^ mg-protein^−1^, with SOD activities between 0.4 and 6.8 U mg-protein^−1^ [47]. In contrast, moderately tolerant anaerobes (e.g. *Propionibacterium acnes* and *Bacteroides vulgatus*), which survived 4 to 8 h of oxygen exposure, showed higher SORRs (14.7–22.9 nmol-O_2_ min^−1^ mg-protein^−1^) and moderate SOD activities (7.0–12.5 U mg-protein^−1^).

These observations suggest that ROS generated during oxygen reduction may impose greater oxidative stress on anaerobic microorganisms with low SOD activity and high SORR. Consequently, obligate anaerobes with similarly low SOD activity but slower SORR may exhibit greater survival under microoxic conditions. Taken together, although *Scalindua* exhibits a relatively high SORR, its exceptionally high SOD activity indicates that it possesses sufficient capacity to withstand O₂ exposure.

### Oxygen consumption mechanisms in *Scalindua* sp. biomass

A central and compelling question is: who within the *Scalindua* sp. biomass is responsible for oxygen consumption? Although the *Scalindua* sp. biomass used in this study was highly enriched (>98%), it still contained a minor fraction (~2%) of coexisting bacteria. Is oxygen removal primarily mediated by the dominant *Scalindua* sp., or by the minority cohabitants? If *Scalindua* sp. itself is responsible, what molecular or physiological mechanisms enable this process? The following discussion provides a careful examination of these questions.

Taken together, multiple lines of evidence strongly support that *Scalindua* sp. is the primary driver of oxygen consumption: (i) high expression of key oxygen-reducing genes including cbb₃-type CcO and A-type flavodiiron proteins (FDPs), was consistently detected in *Scalindua*; (ii) batch experiments with specific inhibitors (e.g. ClO₃^−^, NaN₃) demonstrated a direct coupling between oxygen consumption, NXR-mediated nitrite oxidation, and the activity of *Scalindua*’s cbb₃-type CcO; (iii) CcO activity closely paralleled both the transcriptional dynamics of CcO subunit genes and the substrate-dependent variation in SORRs; (iv) coexisting bacteria, representing only ~2% of the community, lack nitrite-oxidizing capacity and are sensitive to applied inhibitors (e.g. penicillin G), making their contribution unlikely; and (v) their biomass abundance is insufficient to account for the observed oxygen consumption, which far exceeds canonical aerobic NO₂^−^ oxidation rates.

Each inhibitor used in this study has known off-target effects that may influence cellular physiology under microoxic conditions. Acetylene can affect heme-containing oxidases; methanol may alter redox balance; PTIO can generate reactive nitrogen species (RNS) during redox cycling; and azide broadly inhibits heme-copper oxidases and other metalloenzymes. Although these off-target actions should be considered when interpreting inhibitor assays, they do not affect the main conclusions of this study.

### O_2_ consumption by *Scalindua* sp.

**Figure 10:**
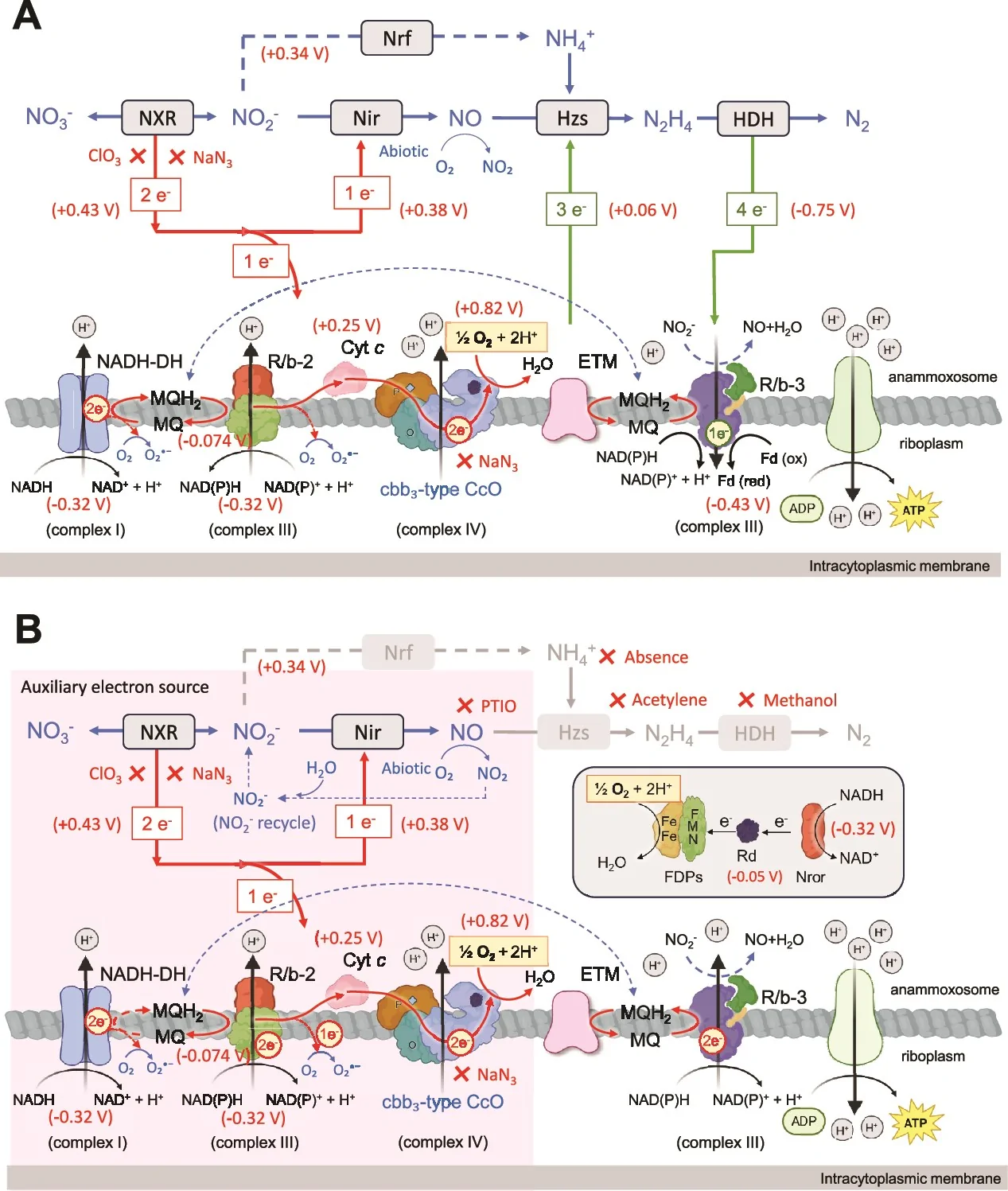
Schematic overview of electron flow and the associated oxygen scavenging mechanisms proposed for *Scalindua* sp., under conditions with both NH₄^+^ and NO₂^−^ (A), and with NO₂^−^ alone (B). *Scalindua* sp. harbors membrane-bound respiratory complexes, including complex I (NADH dehydrogenase), complex III (Rieske/cytochrome *b*-type), and complex IV (cbb_3_-type CcO). Standard midpoint redox potentials at pH 7 (*E*_0_’) are indicated in red within parentheses. The number of electrons transferred in each reaction is indicated in the red and green boxes. When both NH_4_^+^ and NO_2_^−^ are supplied, the core anammox reaction (NH₄^+^ + NO → N₂H₄ → N₂) proceeds (A). Although hydrazine (N₂H₄) oxidation can supply low-potential electrons, these electrons were most likely not directed toward oxygen reduction but instead allocated to ferredoxin reduction for carbon fixation. Because oxygen reduction by CcO withdraws electrons from the cyclic electron flow, these electrons must be replenished through NO₂^−^ oxidation to NO₃^−^ by NXR, or alternatively via other available electron donors such as NAD(P)H. Notably, oxygen reduction persists solely in the presence of NO_2_^−^, even when anammox activity (i.e. N₂ production) is inhibited by anammox-specific inhibitors including acetylene, methanol, and PTIO, or under NH_4_^+^-absent conditions (B). Nitrite is oxidized to nitrate (NO₃^−^; *E*_0_' = +0.43 V) via NXR, generating high-potential electrons that are transferred either toward oxygen reduction (O₂ → H₂O;*E*_0_'  = +0.82 V) via cbb₃-type CcO and ROS formation, or toward NO_2_^−^ reduction to NO (NO; *E₀*′ = +0.38 V) via Nir. When NH₄^+^ is absent (B), NO₂^−^ oxidation appears to function as a major electron source for oxygen reduction, although the underlying electron-transfer mechanism remains unresolved. This electron allocation reflects a thermodynamically favorable strategy that supports metabolic flexibility under microoxic conditions. cyt *c*, cytochrome *c*; CcO, cytochrome *c* oxidase; NADH-DH, NADH dehydrogenase; NXR, nitrite:nitrate oxidoreductase; Nir, nitrite reductase; Hzs, hydrazine synthase; HDH, hydrazine dehydrogenase; Nrf, formate-dependent nitrite reductase forming ammonium; MQ, menaquinone-7; ETM, electron transport module; R/b-2 and R/b-3, novel Rieske/cytochrome *b* complexes-2 and -3, respectively. This was partly adapted with reference to references [54, 55, 64].

RT-qPCR analyses demonstrated high expression of key oxygen-reducing genes in *Scalindua* sp. biomass, notably those encoding cbb₃-type CcO and A-type FDPs (Fig. 6). A-type FDPs are predominantly found in anaerobic bacteria and archaea [48, 49, 50], and possess a non-heme diiron center and a flavin mononucleotide moiety. These structural features enable electron acceptance from reduced rubredoxin (Rd_red_), which channels NADH-derived electrons via NADH:rubredoxin oxidoreductase (Nror) to FDPs, thereby driving the complete reduction of O₂ to H₂O or NO to N₂O without producing ROS/RNS [51, 52, 53] (Fig. 6D). Accordingly, A-type FDPs and CcO likely function as critical oxygen-scavenging systems, safeguarding oxygen-sensitive metabolic processes including those mediated by Rieske iron–sulfur proteins. Genomic analyses indicate that *Scalindua* encodes homologs of Rieske [2Fe-2S] proteins and cytochrome *b* subunits, key components of the cytochrome *bc*₁ complex (respiratory complex III) [13, 54, 55], which may facilitate menaquinone (MQ) oxidation and contribute to electron transport (Fig. 10).

Integrating the distinct transcriptional patterns of genes encoding CcO, FDP, and SOD with direct measurements of CcO enzymatic activity suggests that these three proteins play complementary roles in oxygen scavenging. CcO is constitutively expressed at high levels and likely serves as the primary oxygen-scavenging mechanism. In the OMZ environments where *Scalindua* resides, small-scale oxygen intrusions occur frequently enough that maintaining active CcO is selectively advantageous, even under predominantly anoxic conditions. This constitutive expression enables *Scalindua* to respond rapidly to transient oxygen exposure, consistent with the immediate onset of oxygen consumption observed in our experiments. This interpretation is strongly supported by CcO activity assays, which showed a rapid three-fold increase in CcO activity within 15 min after substrate addition, followed by stabilization at approximately twice the pre-addition level after 1.5 h (Fig. 9). These dynamics closely paralleled the transcriptional responses of CcO subunit genes (Fig. 8E). In contrast, FDP exhibits strong oxygen inducibility and may function as an auxiliary detoxification pathway when CcO becomes saturated under conditions of limited electron availability. Although SOD displays relatively low transcript abundance, its strong oxygen inducibility and high enzymatic activity suggest that substantial amounts of SOD protein are constitutively present in the cells. The pronounced transcriptional induction observed after oxygen exposure therefore likely reflects reinforcement or activation of existing protein pools rather than large-scale *de novo* synthesis. Together, these lines of evidence reconcile the mismatch between gene expression and enzyme activity and highlight the multi-layered architecture of oxygen-tolerance mechanisms in *Scalindua* sp.

### Cytochrome *c* oxidase in *Scalindua* sp.

The cbb₃-type CcO is a high-affinity terminal oxidase that catalyzes the four-electron reduction of molecular oxygen to water without generating ROS (O₂ + 4e^−^ + 4H^+^ → 2H₂O, *E*₀′ = +0.82 V). In addition, CcO can contribute to proton-gradient formation by intrinsic proton-pumping activity and thus may offer an energetic advantage under microoxic conditions. The CcO is a multimeric respiratory complex comprising the catalytic subunit CcoN, the electron-transfer subunits CcoO and CcoP, and the accessory factor CcoQ. *Scalindua* sp. encodes homologs of *ccoN*, *ccoO*, and *ccoP*, but no clear homolog of *ccoQ* has been identified in currently available genomes [26], and these genes do not form a canonical ccoNOQP operon [56, 57]. In many bacteria, the *cco* genes are induced under low-oxygen conditions and regulated by oxygen-responsive transcriptional regulators [58, 59, 60, 61].

Unlike most bacteria, *Scalindua* shows strong nitrite-dependent induction of its *cco* genes (Fig. 7E). A similar regulatory pattern has also been reported for *Pseudomonas aeruginosa* [61]. Consistent with this transcriptional pattern, CcO enzymatic activity in *Scalindua* also increases markedly in the presence of nitrite (Fig. 9), indicating that both CcO expression and activity are stimulated by nitrite-dependent signaling rather than by oxygen limitation, even though the overall electron balance cannot be fully explained by nitrite oxidation alone. Although the underlying mechanism remains unresolved, the reduction of nitrite to NO in anammox metabolism suggests that NO, rather than NO_2_^−^ itself, may act as the primary signal triggering CcO expression and activity. NO- and oxygen-responsive transcription factors commonly use Fe-S clusters as sensory modules [62, 63], and NO-induced modifications of these clusters could mediate the transcriptional regulation of *cco* genes.

Overall, our results suggest that the cbb₃-type CcO in *Scalindua* is likely expressed constitutively, keeping the oxygen-reduction machinery in a primed state that enables a rapid response to oxygen exposure. Its activity appears to be modulated by NO_2_^−^ availability, potentially through both electron supply and NO-linked redox signaling. During oxygen reduction, this oxidase may translocate protons across the membrane, thereby contributing to a PMF that could support ATP synthesis. However, the quantitative importance of this pathway for cellular energy conservation remains unresolved and warrants further investigation.

### Proposed electron transport pathway

Under anammox conditions where both NH_4_^+^ and NO_2_^−^ are supplied, potential electron donors for oxygen reduction by cbb₃-type CcO include low-potential electrons derived from hydrazine (N₂H₄) oxidation (N_2_H_4_ → N_2_ + 4H^+^ + 4e^−^, *E*_0_'  = −0.75 V) and high-potential electrons derived from nitrite oxidation to nitrate (NO₂^−^ → NO₃^−^ + 2H^+^ + 2e^−^, *E*_0_' = +0.43 V) [64] (Fig. 10A). The low-potential electrons are thermodynamically favorable to be allocated to ferredoxin reduction for carbon fixation, as CO₂ reduction requires a much lower redox potential (*E*_0_’ = −0.43 V) [28, 64]. In contrast, the high-potential electrons are sufficient to replenish cyclic electron flow and expected to be delivered to the oxidase via reduced cytochrome *c*, specifically its ferrous form, ferrocytochrome *c* [29, 54]. This electron allocation provides metabolic flexibility under microoxic conditions, enabling concurrent anammox activity and oxygen reduction. However, when NH₄^+^ is absent or hydrazine production and oxidation are inhibited by acetylene or methanol, nitrite and other electron donors such as NAD(P)H could provide auxiliary electrons for cbb₃-type CcO-mediated oxygen reduction (Fig. 10B). In NO_2_^–^ only addition incubations, we observed coordinated increases in CcO activity, transcription of CcO genes, and oxygen consumption. These observations suggest that nitrite availability modulates CcO-mediated oxygen reduction through regulatory mechanisms independent of enhanced nitrite oxidation. This flexibility implies that *Scalindua* can mitigate oxidative stress by dynamically reorganizing its electron-transfer pathways.

### *Rhizobiales* as a potential O_2_ scavenger?

To date, *Rhizobiales* genera such as *Bradyrhizobium* and *Rhizobium* have not been shown to exhibit nitrite-oxidizing activity comparable to canonical NOB like *Nitrospira* and *Nitrobacter*. Instead, *Rhizobiales* are primarily associated with reductive nitrogen transformations, including nitrogen fixation and denitrification [65, 66, 67]. Moreover, our prior study demonstrated complete inhibition of *Rhizobiales* ureolytic activity by 500 mg l^−1^ of penicillin G [68], which was applied in all batch incubations in the present study. Thus, *Rhizobiales* activity was likely suppressed, and their contribution to oxygen consumption minimal. Furthermore, the negligible biomass of taxa ranked below the top three, including *Rhizobiales*, renders them unlikely contributors to the observed oxygen consumption. Together, these results provide strong support that oxygen consumption within the *Scalindua* sp. biomass is overwhelmingly driven by *Scalindua* itself.

### Possibility of abiotic NO-dependent O_2_ reduction

If nitrite oxidation to nitrate (NO₂^−^ + 0.5 O₂ → NO₃^−^ + 2e^−^; *E*₀′ =+0.43 V) via NXR serves as an auxiliary electron source for oxygen reduction mediated by cbb₃-type CcO, a fraction of the generated electrons may be diverted to nitrite reduction to NO (2NO_2_^−^ + 2e^−^ + 2H^+^ → 2NO + H_2_O; *E*₀′ = +0.38 V) (Fig. 10). In this context, anammox bacteria are known to disproportionate NO_2_^−^ into NO_3_^−^ and NO, as represented by the following reaction [28, 55, 69]:


(1) \begin{equation*} {{3\mathrm{NO}}_2}^{-}+{2\mathrm{H}}^{+}\to 2\mathrm{NO}+{{\mathrm{NO}}_3}^{-}+{\mathrm{H}}_2\mathrm{O} \end{equation*}

Nitrite disproportionation plays a central role in anammox metabolism by simultaneously supplying NO, a key intermediate in the anammox pathway (NH₄^+^ + NO → N₂H₄ → N₂ + H₂O). Previous studies employing DAF-2 DA have demonstrated that NO is generated during the anammox reaction pathway [70], and that its elimination by PTIO leads to inhibition of the anammox reaction [71]. Once produced, NO rapidly reacts with molecular oxygen to form nitrogen dioxide (2 NO + O₂ → 2 NO₂), which subsequently hydrolyzes to nitric and nitrous acids (2 NO₂ + H₂O → HNO₃ + HNO₂). Thus, NO can function as a potential abiotic oxygen scavenger.

Nitrite disproportionation would theoretically yield a stoichiometric ratio of NO₂^−^ consumed, NO produced (PTIO consumed), and NO₃^−^ produced (ΔNO₂^−^: ΔNO (PTIO): ΔNO₃^−^) of 1: 0.67: 0.33. This ratio aligns with the experimentally observed value (1: 0.48: 0.27) in NO₂^−^ only addition experiments with PTIO (Fig. 5B), providing strong evidence that *Scalindua* disproportionates NO₂^−^ into NO₃^−^ and NO. The observed stoichiometric produced NO (consumed PTIO) and consumed O_2_ ratios (ΔNO (PTIO): ΔO_2_) were however 1: 6.8, which significantly diverges from the theoretical ratio of 1: 0.5 for complete NO-based oxygen removal. In addition, despite the rapid and specific elimination of NO by PTIO [43, 71], SORR was only moderately reduced (~18% lower than control without PTIO, *P* < .05) (Figs 5 and 8), suggesting limited abiotic NO-based oxygen removal. Our findings demonstrate that nitrite disproportionation contributes partially; however, this pathway alone is insufficient to account for the oxygen consumption observed.

### Electron balance

In the NO₂^−^ only addition incubations, the average ΔNO₂^−^: ΔO₂:ΔNO_3_^−^ ratio of 1: 2.8: 0.3 deviated markedly from the canonical 1: 0.5: 1 expected for aerobic nitrite oxidation (NO₂^−^ + 0.5 O₂ → NO₃^−^), indicating that nitrite was not primarily oxidized via the canonical aerobic pathway (Fig. 5A). In the NO₂^−^ only addition incubations with PTIO, the average ΔNO₂^−^: ΔO₂: ΔNO (PTIO): ΔNO₃^−^ ratio was 1: 6.8: 0.48: 0.28 (Fig. 5B), again showing that nitrite consumption was substantially lower than oxygen removal. These disproportionately high oxygen consumptions cannot be explained by nitrite oxidation alone and indicate that the electron balance does not fully close.

Several factors may account for this discrepancy. In the absence of NH₄^+^, anammox proceeds without carbon fixation via ferredoxin reduction, and electrons derived from nitrite oxidation are likely recycled internally rather than used for net substrate conversion. Additional electron input may also arise from NAD(P)H generated through processes such as glycogen degradation. Oxygen can undergo not only four-electron reduction to water via CcO but also one-electron reduction to ROS, which are subsequently disproportionated by SOD and CAT to H₂O₂ and ultimately H₂O, thereby increasing overall O₂ consumption. Furthermore, NO produced through nitrite disproportionation reacts rapidly with O₂ to form NO₂, which hydrolyzes back to NO₂^−^ and NO₃^−^ under typical anammox cultivation conditions, establishing a “NO₂^−^ cycling” loop (NO₂^−^ → NO → NO₂^−^/NO₃^−^) that may lead to underestimation of net nitrite consumption. In addition to these biochemical factors, nitrite may modulate CcO-mediated oxygen reduction through unresolved NO-dependent signaling rather than enhanced nitrite oxidation alone. Given that electron-transfer pathways in anammox bacteria remain only partially resolved, a complete electron balance cannot be achieved in the present study.

### Ecological implications

Although the bioreactor biomass exhibited rapid oxygen consumption and measurable oxygen tolerance, these traits must be interpreted in the context of natural assemblages. In seawater, *Scalindua* typically occurs at far lower abundances than in our enrichment culture, and *in situ* O₂ consumption rates are therefore expected to be much lower—consistent with reports that only a few μM O₂ can inhibit anammox activity in natural OMZs [10, 21]. Even so, intermittent O₂ intrusions and microscale O₂ heterogeneity in OMZs may still select for the retention of O₂-detoxification and tolerance mechanisms, even if anammox activity itself is largely suppressed under oxic conditions.

To avoid overgeneralization, we note that the ~5 μM DO threshold observed in this study reflects the specific conditions of our short-term, well-mixed batch incubations using planktonic enriched anammox cultures. These operational thresholds are influenced by the homogeneous DO distributions and gas–liquid exchange dynamics inherent to the experimental setup. In natural OMZs, however, anammox bacteria may occur within aggregates or particle-associated communities, where diffusion limitation and steep microscale DO gradients can create anoxic microenvironments even when bulk DO remains several micromolar. Such physical structuring may shift the effective *in situ* DO threshold for anammox activity. Thus, the ~5 μM value should be viewed as a laboratory-derived benchmark rather than a universal threshold for OMZ systems.

## Conclusion

Under microoxic conditions, *Scalindua* sp. actively removed DO at rates of ~10 nmol-O₂ min^−1^ mg-protein^−1^, most likely via oxygen-reducing enzymes such as cbb₃-type CcO, with minimal ROS generation. Upon oxygen depletion to ~5 μM, anammox activity, evidenced by ^29^N₂ production, resumed immediately. Nitrite availability was essential for sustaining oxygen consumption. The pronounced induction of CcO activity and the transcription of CcO subunit genes upon nitrite addition further may suggest that nitrite functions not only as an auxiliary electron donor but also as a regulatory signal that activates CcO-mediated oxygen reduction. *Scalindua* sp. exhibited moderate oxygen-reduction rates coupled with elevated SOD activity, enabling rapid and complete detoxification of ROS and conferring both high and reversible oxygen tolerance. Collectively, these findings demonstrate that *Scalindua* mitigates oxidative stress by flexibly redirecting electron flow, highlighting a substrate-driven oxygen-detoxification mechanism that operates uncoupled from core anammox metabolism under transiently microoxic conditions.

## Supplementary Material

SI-Oxidative_defense_strategies-Revised_Final_wrag075

## Data Availability

All data generated or analysed during this study are included in this published article and its supplementary information files.
