# Supplementary material for: Oxygen scavenging enables microoxic survival of the marine anammox bacterium Scalindua sp
Source: ISME J. 2026 Apr 2;20(1):wrag075. doi: 10.1093/ismejo/wrag075 (PMC13253579; doi:10.1093/ismejo/wrag075)
Supplement: SI-Oxidative_defense_strategies-Revised_Final_wrag075 [file si-oxidative_defense_strategies-revised_final_wrag075.docx]

**Supplemental Information**

**Oxygen scavenging enables microoxic survival of the marine anammox bacterium *Scalindua* sp.**

By

**Satoshi Okabe^1 *^, Keishi Nukada^2^, Keitaro Horiguchi^2^, and Mamoru Oshiki^1^**

*^1^ Division of Environmental Engineering, Faculty of Engineering, Hokkaido University, North 13, West 8, Sapporo, Hokkaido 060-8628, Japan.*

*^2^ Division of Environmental Engineering, Graduate School of Engineering, Hokkaido University, North 13, West 8, Sapporo, Hokkaido 060-8628, Japan.*

*^*^Corresponding author: Satoshi Okabe*

*E-mail: sokabe@eng.hokudai.ac.jp.*

This file contains

- Text
- Table S1
- Figures S1 – S11

**Microbial Community Analysis**

Biomass samples were obtained from two membrane bioreactors (MBRs) enriched with Scalindua and subjected to Percoll density gradient centrifugation. Genomic DNA was extracted by adding Lysis Solution F (Nippon Gene) to the samples, followed by mechanical disruption using a Shake Master Neo (BMS) at 1,500 rpm for 2 minutes. The lysates were centrifuged at 12,000 *×g* for 2 minutes, and DNA was purified with the Lab-Aid 824s DNA Extraction Kit (ZEESAN).

Amplicon libraries targeting the V4 region of the 16S rRNA gene were prepared using a two-step PCR protocol. The first PCR employed primer sets 1st-515f MIX (ACACTCTTTCCCTACACGACGCTCTTCCGATCT-NNNNN-GTGCCAGCMGCCGCGGTAA) and 1st-806rb MIX (GTGACTGGAGTTCAGACGTGTGCTCTTCCGATCT-NNNNN-GGACTACNVGGGTWTCTAAT). The second PCR used indexed primers 2ndF (AATGATACGGCGACCACCGAGATCTACAC-Index2-ACACTCTTTCCCTACACGACGC) and 2ndR (CAAGCAGAAGACGGCATACGAGAT-Index1-GTGACTGGAGTTCAGACGTGTG). Library quality was assessed with a Fragment Analyzer (Agilent Technologies) using the dsDNA 915 Reagent Kit.

Sequencing was performed on the Illumina MiSeq platform with the MiSeq Reagent Kit v3 in a 2 × 300 bp paired-end configuration. Raw reads were processed using the FASTX Toolkit (ver. 0.0.14). Reads with exact matches to primer sequences were extracted using the fastx barcode splitter tool, and primer sequences were removed with fastx trimmer. Low-quality reads (Phred score < 20) and paired reads shorter than 130 bp were discarded using Sickle (ver. 1.33). Paired-end reads were merged with FLASH (ver. 1.2.11) under the following parameters: minimum overlap = 10 bp, read length = 230 bp, and merged length = 250 bp.

Denoising and chimera removal were conducted using the DADA2 plugin in QIIME2 (ver. 2023.7), resulting in representative sequences and an ASV table. Taxonomic classification was performed with the feature-classifier plugin in QIIME2 using the Greengenes database (ver. 13.8, 97% OTU reference).

**RT-qPCR protocol for transcriptional analysis**

*Scalindua* cells exposed to oxygen for 1 h and 24 h were collected for total RNA extraction using the RNeasy PowerFecal Pro Kit (QIAGEN). DNA contamination was removed with the TURBO DNA-free Kit (Thermo Fisher Scientific), and reverse transcription was performed with the High Capacity cDNA Reverse Transcription Kit with RNase Inhibitor (Thermo Fisher Scientific).

To confirm successful cDNA synthesis, PCR was conducted for 30 cycles using SapphireAmp® Fast PCR Master Mix (Takara Bio), and products were analyzed by agarose gel electrophoresis (1.5% gel, 100 V, 30 min) with fluorescent staining. Control PCR reactions using RNA without reverse transcription were performed to assess DNA contamination.

qPCR reactions contained 5 µL KAPA SYBR Fast qPCR Master Mix, 2 µL of a 1 µM primer mix, 0.2 µL ROX Low reference dye (50×), 1.8 µL DNA-free water, and 1 µL cDNA template. The thermal cycling protocol consisted of an initial denaturation at 95°C for 3 min, followed by 40 cycles of 95°C for 3 s and 60°C for 30 s.

Gene-specific primers were designed for putative oxygen detoxification genes, including *fdp1* (SCALA7_01450), *fdp2* (SCALA7_11410), *fdp3* (SCALA7_19040), *fdp4* (SCALA7_23040), *fdp-rd* (SCALA7_01460), *kat* (SCALA7_02240), *rbr1* (SCALA7_05050), *rbr2* (SCALA7_36590), *nlr/sor* (SCALA7_12610), *sod* (SCALA7_17830), *gpx1* (SCALA7_18270), *gpx2* (SCALA7_22180), *ccp* (SCALA7_32150), and the cbb₃-type cytochrome c oxidase subunits *ccoP*, *ccoO*, and *ccoN* (SCALA7_32500, SCALA7_32510, and SCALA7_32520, respectively) (see **Table S1** for details). Standard curves were generated using tenfold serial dilutions of plasmid DNA containing the corresponding gene fragments, and PCR efficiency was calculated from the slope of each curve. Initial cDNA quantities were estimated from *Ct* values and amplification efficiency. Expression levels were normalized to hydrazine dehydrogenase (*hdh*, SCALA7_07650), which is stably expressed under anoxic conditions.

|  | **Table S1** RT-qPCR primer sets used to quantify expression levels of putative oxygen detoxification genes. The gene encoding hydrazine dehydrogenase (*hdh*) served as the process control. | | | | | |
| --- | --- | --- | --- | --- | --- | --- |
| No | Locus_tag | Hypothetical gene | Protein | Sequence (5’ to 3’) | | PCR amplicon  (bp) |
| 1 | SCALA7_01450 | *fdp1* | homologous to the flavodiiron protein of *Desulfovibrio vulgaris* | Forward | TGCATCAGTAATGGGCAAAA | 103 |
|  |  |  |  | Reverse | TTTGCTACCGAGGCTCAACT |  |
| 2 | SCALA7_01460 | *fdp-rd* | a flavodiiron-rubredoxin fusion protein | Forward | GCAATTGGCGATCCAGAC | 100 |
|  |  |  |  | Reverse | AGCTTTCTTTTCCAGCACCA |  |
| 3 | SCALA7_02240 | *kat* | Catalase | Forward | GTGGCAGGAACAACCTGATT | 170 |
|  |  |  |  | Reverse | CCATTGCTCGATCGGTATTT |  |
| 4 | SCALA7_05050 | *rbr1* | homologous to the rubrerythrin of *Desulfovibrio vulgaris* | Forward | ATTTGCCAAAATCGCAAAAG | 108 |
|  |  |  |  | Reverse | TCGGCAAGATCCTTATACCG |  |
| 5 | SCALA7_11410 | *fdp2* | homologous to the flavodiiron protein of *Clostridium difficile* 630 | Forward | CGGGTCACGGACCTATTCTA | 144 |
|  |  |  |  | Reverse | CATGTGCCATTTTCTTGGTG |  |
| 6 | SCALA7_12610 | *nlr/sor* | Neelaredoxin/superoxide reductase | Forward | CCTCCGGTAGTGAAGACAGG | 126 |
|  |  |  |  | Reverse | CCCAAGATAGACCGATCCAA |  |
| 7 | SCALA7_17830 | *sod* | Mn/Fe-type superoxide dismutase | Forward | GGCTTTTCAAACAGCAGGAG | 164 |
|  |  |  |  | Reverse | AATACGCATGTTCCCACACA |  |
| 8 | SCALA7_18270 | *gpx1* | Glutathione peroxidase | Forward | GGGAGAGTGCTCTTCATCGT | 157 |
|  |  |  |  | Reverse | TATCTGTTCCCGGTTCTTGG |  |
| 9 | SCALA7_19040 | *fdp3* | homologous to the flavodiiron protein of *Clostridium difficile* 630 | Forward | TCGTACCGACAGAACATGGA | 171 |
|  |  |  |  | Reverse | CAGGTTCGGTGTGATTGATG |  |
| 10 | SCALA7_22180 | *gpx2* | Glutathione peroxidase | Forward | AATACTGCCTCCGAATGTGG | 125 |
|  |  |  |  | Reverse | GGCTCTTGTTTTCCGAACTG |  |
| 11 | SCALA7_23040 | *fdp4* | homologous to the flavodiiron proteins of *Clostridium difficile* 630 and *Desulfovibrio vulgaris* | Forward | GCTCACACTTTTGCGATGAA | 124 |
|  |  |  |  | Reverse | TTTTCCATTGCCTTGAGGAC |  |
| 12 | SCALA7_32150 | *ccp* | Cytochrome *c* peroxidase | Forward | TAGCCCTTGCCATTATCACC | 169 |
|  |  |  |  | Reverse | CTGAGCGGTATTGGTTGGAT |  |
| 13 | SCALA7_32500 | *ccoP* | cbb3-type cytochrome *c* oxidase subunit P | Forward | ATCGCACACACCATCAGGTA | 147 |
|  |  |  |  | Reverse | ACCCTCAGGCTCTTTTGGAT |  |
| 14 | SCALA7_32510 | *ccoO* | cbb3-type cytochrome *c* oxidase subunit O | Forward | TTTTCGGTCTTCCTCGGTTA | 101 |
|  |  |  |  | Reverse | GTGGGATCCTCCTCATGAAA |  |
| 15 | SCALA7_32520 | *ccoN* | cbb3-type cytochrome *c* oxidase subunit N | Forward | TTTGCGGGGATCTATCACTC | 153 |
|  |  |  |  | Reverse | CGCGTACTCTTTCCCTTGAG |  |
| 16 | SCALA7_36590 | *rbr2* | homologous to the rubrerythrin of *Clostridium difficile* 630 | Forward | TGCAGTGAACGCAAACTTTC | 120 |
|  |  |  |  | Reverse | ATGGCGATTGATTTCAGGAC |  |
| 17 | SCALA7_07650 | *hdh* | Hydrazine dehydrogenase | Forward | CGAGACACACCTTTTCAGCA | 202 |
|  |  |  |  | Reverse | TGACAGGTTGGTGTTCGGTA |  |

**Figure S1**. **Taxonomic assignments of the enriched *Scalindua* sp. used in this study.** The phylogenetic tree was generated using AnnoTree (Mendler et al., 2019). ANI scores between the anammox bacterial genomes shown in this figure are available in our previous work (Oshiki et al., 2022). The scale bar represents 10% sequence divergence. This tree was modified from the previous publication (Okabe et al., 2024).

1. **Mendler, K., Chen, H., Parks, D. H., Lobb, B., Hug, L. A., Doxey, A. C.,** 2019. AnnoTree: visualization and exploration of a functionally annotated microbial tree of life. Nuc Acids Res; 47, 4442–4448.

2. **Oshiki, M., Takaki, Y., Hirai, M., Nunoura, T., Kamigaito, A., Okabe, S.** 2022. Metagenomic analysis of five phylogenetically distant anammox bacterial enrichment cultures. Microb. Environ. 37, ME22017.

3. **Okabe, S., Kamizono, A., Zhang, L., Kawasaki, S., Kobayashi, K., Oshiki, M.** 2024. Salinity tolerance and osmoadaptation strategies in four genera of anammox bacteria: *Brocadia*, *Jettenia*, *Kuenenia*, and *Scalindua*. Environ Sci Technol. **58**(12), 5357-5371.

**Effect of Percoll density separation on anammox activity**

**
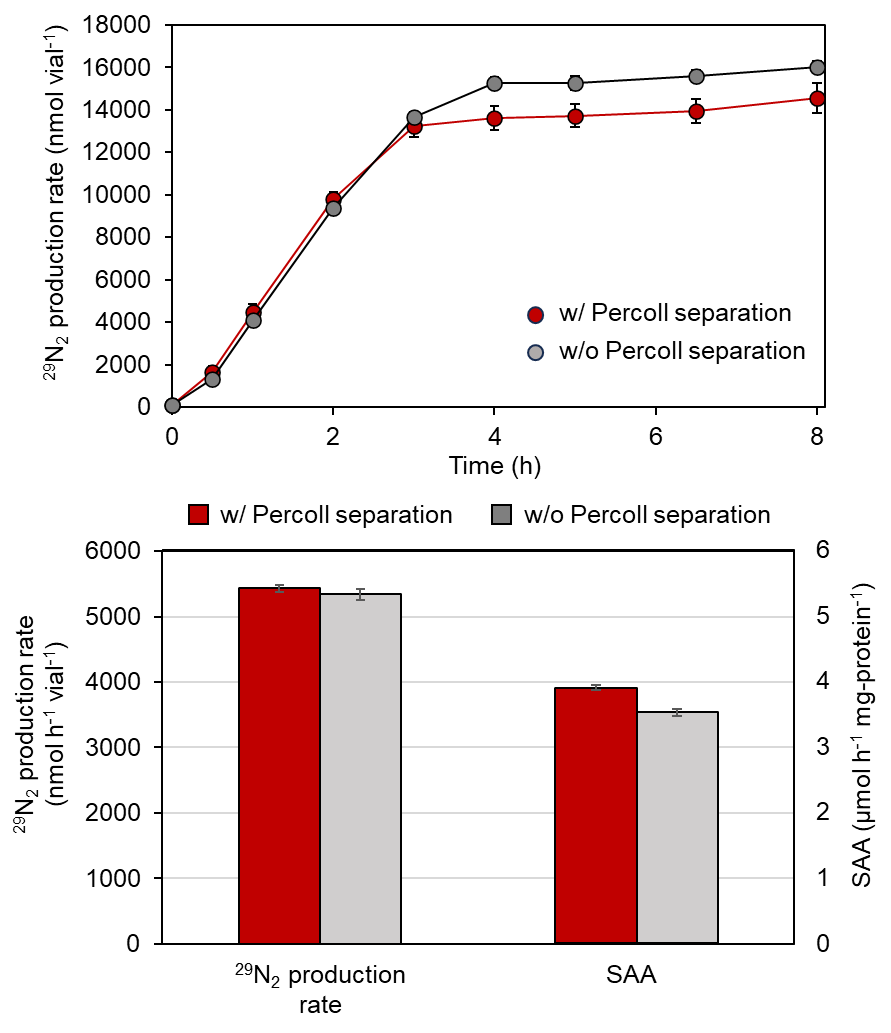
**

**Figure S2 Effect of Percall separation on anammox activity (^29^N_2_ production rate and specific anammox activity, SAA).** The ²⁹N₂ production rate and SAA did not differ significantly between treatments with and without Percoll separation (n=3).

**
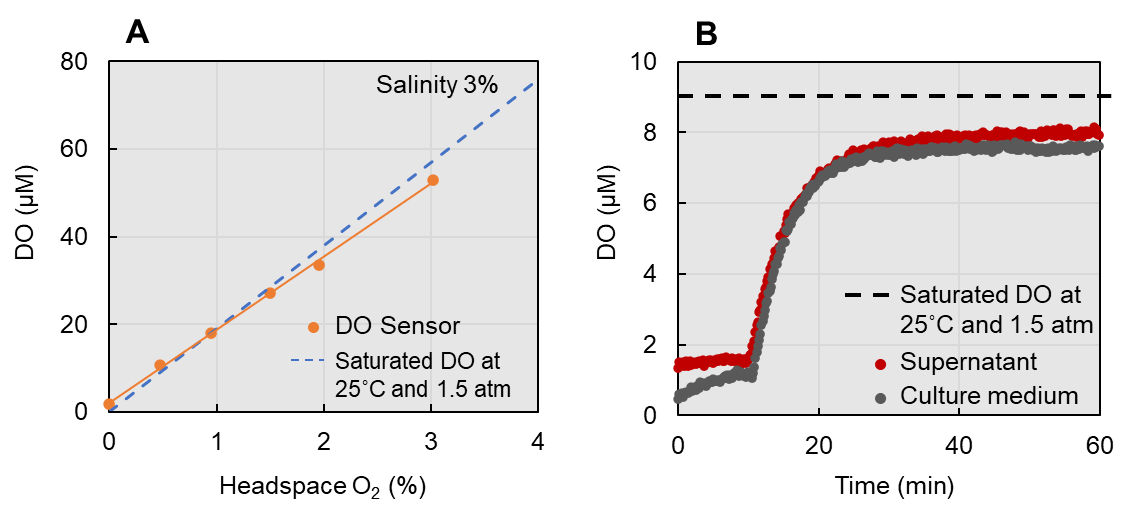
**

**Figure S3. (A) Relationship between injected O₂ and fluorescence-based DO sensor readings at 25 °C and 3% salinity, (B) comparative performance of DO sensors in *Scalindua* culture supernatant versus fresh medium.** Fluorescence-based DO sensors did not interfere with DO measurements in *Scalindua* cultures (salinity 3%).

**Correction method for headspace oxygen concentration (O_2_ %)**

**Experimental procedure**

To evaluate residual oxygen remaining in vials after gas exchange procedures, as well as atmospheric oxygen contamination introduced during GC-MS sample injection, four empty vials were hermetically sealed inside an anaerobic chamber using butyl rubber stoppers and aluminum crimp caps. The headspace of each vial was purged with high-purity helium gas (>99.9%) using a gas exchange apparatus, and the internal pressure was adjusted to 1.5 atm. Each vial was subsequently injected with 0, 25, 50, or 100 µL of high-purity oxygen gas (>99.9%) to achieve defined O₂ concentrations for calibration. The headspace samples (50 µL) were then withdrawn from each vial, injected into the GC/MS to quantify the peak areas corresponding to m/z = 28 (N_2_) and 32 (O_2_), respectively. The calibration was made by plotting the m/z = 32 peak area against the corresponding theoretical headspace O_2_ concentration (%) calculated from the volume of oxygen injected into each vial.

**Results**

GC/MS peak areas for m/z = 32 (O_2_), measured before and after oxygen addition, were plotted against the theoretical headspace oxygen concentration (O_2_ %) estimated from the volume of oxygen injected into each vial (**Fig.** **S4A**). Notably, m/z = 32 signals ranging from approximately 1,000,000 to 1,200,000 were detected even prior to oxygen injection, indicating that oxygen removal via the gas exchange apparatus was incomplete and that residual oxygen remained in the vials. Although the m/z = 32 peak areas exhibited some variability, they showed a strong correlation with the m/z = 28 (N₂) peak areas (**Fig.** **S4B**). When the pre-injection m/z = 32 signal was subtracted from the post-injection signal, the resulting corrected signal values correlated well with the theoretical headspace oxygen concentration (**Fig** **S4C**). These results demonstrate that the residual oxygen concentration in the vial can be estimated by quantifying the O₂ signal of samples without O_2_ addition. Furthermore, the extent of atmospheric oxygen contamination introduced during GC-MS sample injection can also be inferred from the N₂ signal (m/z = 28), even in oxygen-supplemented samples. Thus, temporal changes in the headspace O_2_ concentration (%) in the vial headspace can be corrected by quantifying the increase in the N₂ signal, estimating the atmospheric oxygen contamination introduced during GC/MS measurement, and subtracting it from the observed values.


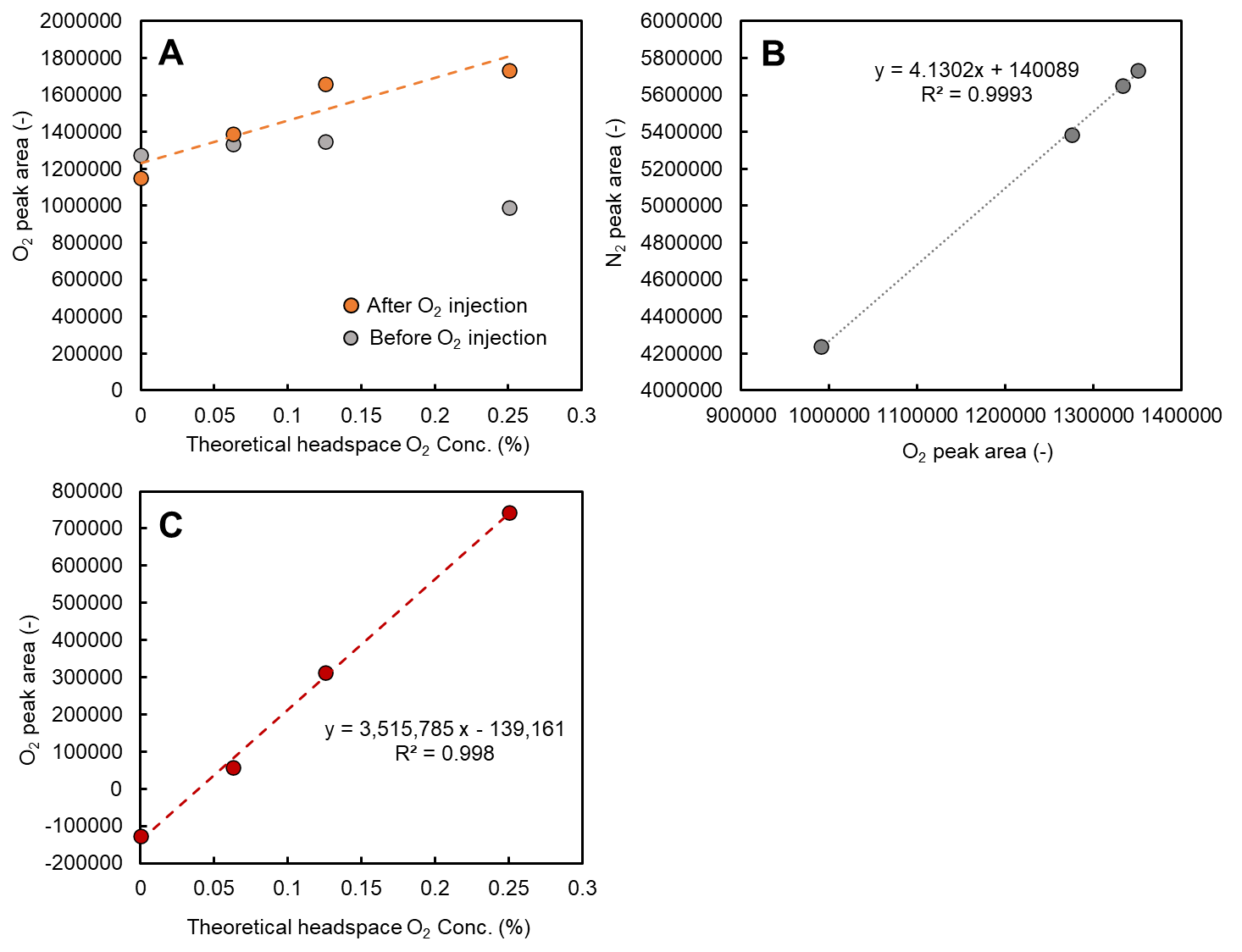


**Figure S4. Correction of oxygen contamination during calibration curve preparation for headspace oxygen quantification by GC/MS.** (**A**) GC/MS peak areas for m/z = 32 (O₂), measured before and after oxygen addition, plotted against the theoretical headspace oxygen concentration (%). (**B**) Correlation between m/z = 32 (O₂) peak areas and m/z = 28 (N₂) peak areas. (**C**) Relationship between corrected m/z = 32 (O₂) peak areas and the theoretical headspace oxygen concentration (%).


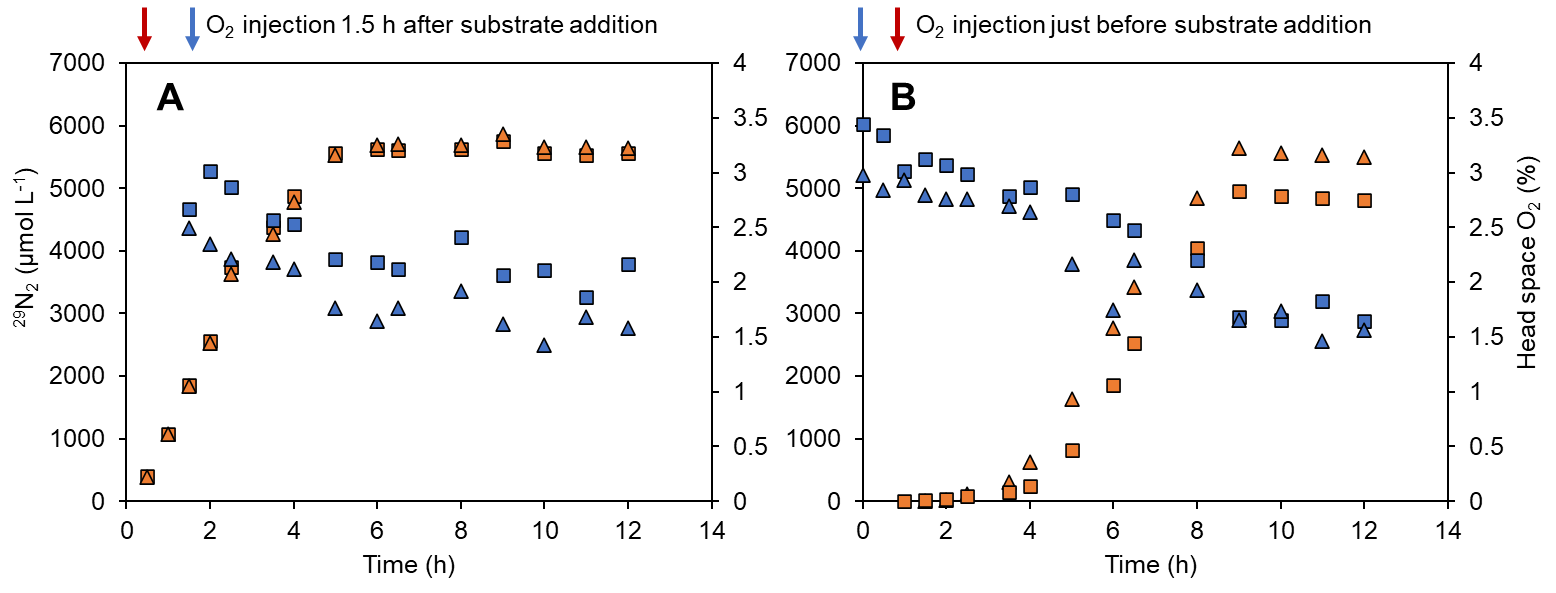


**Figure S5**. **Effect of O_2_ injection on ^29^N_2_ production in the presence and absence of substrate (NH_4_^+^ + NO_2_^-^).** Injecting O₂ during N₂ production does not significantly impact N₂ generation (**A**), whereas introducing O₂ prior to the onset of N₂ production delays its initiation (**B**). Red and blue arrows indicate the addition of substrate (¹⁵NH₄⁺ and ¹⁴NO₂⁻, 5 mM each) and O_2_, respectively. Results from two independent replicates were plotted together in the same graph. One of the three experimental replicates is presented in **Fig. 1** of the main text


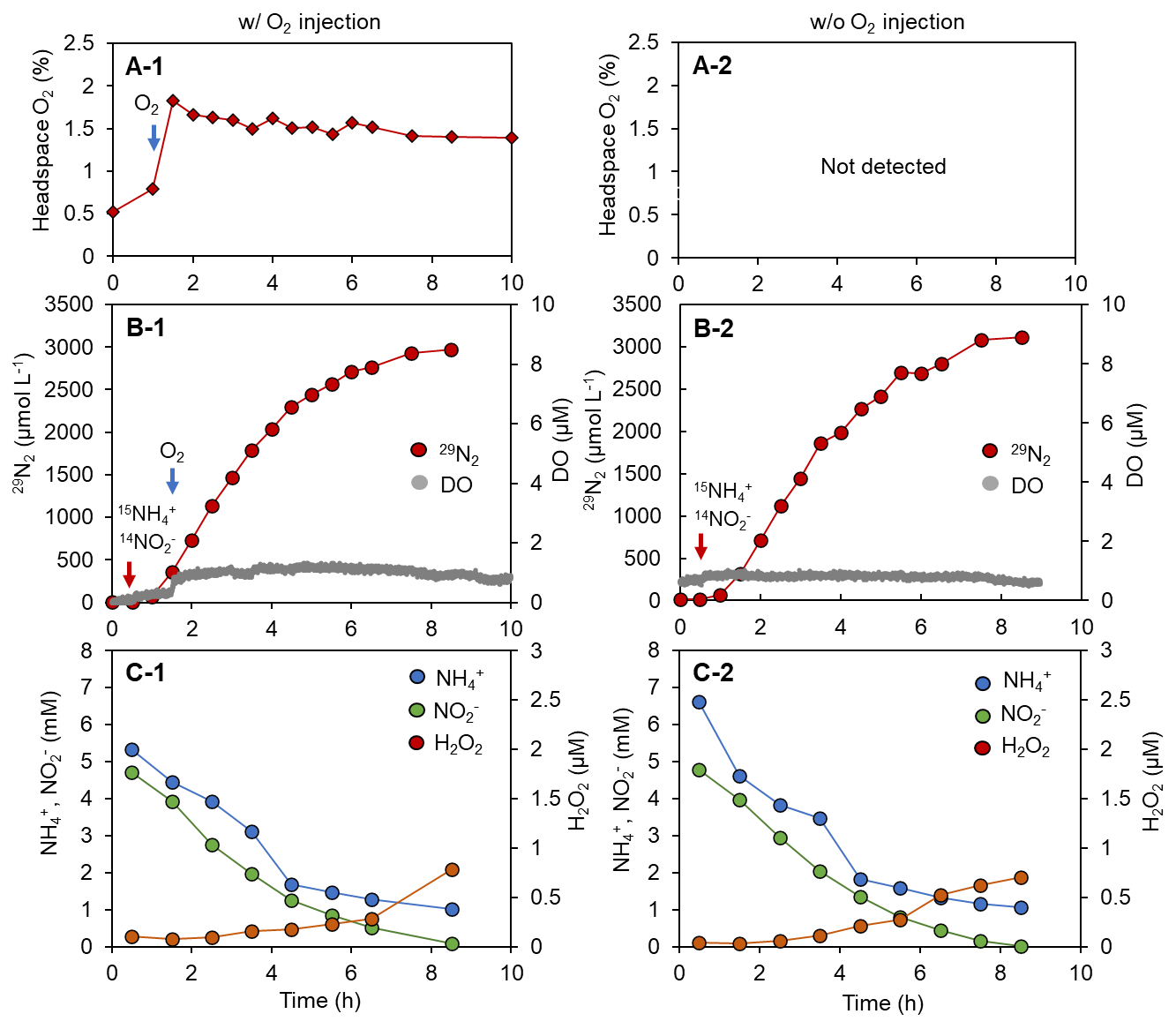


**Figure S6. Extracellular hydrogen peroxide (H₂O₂) accumulation during batch incubations with and without O₂ addition.** Pure oxygen was injected into sealed vials containing culture medium supplemented with anammox substrates (¹⁵NH₄⁺ and ¹⁴NO₂⁻) at 1.5 h (**-1**). Time courses of headspace O₂ concentration (%) (**A**), DO and ²⁹N₂ production (**B**), and extracellular H₂O₂ concentration (**C**) were monitored. In cultures containing *Scalindua* biomass and substrates, DO levels remained stable at low micromolar levels (**B-1**). Extracellular H₂O₂ gradually accumulated during oxygen consumption, but its concentration remained extremely low throughout the incubation (**C-1**).

**
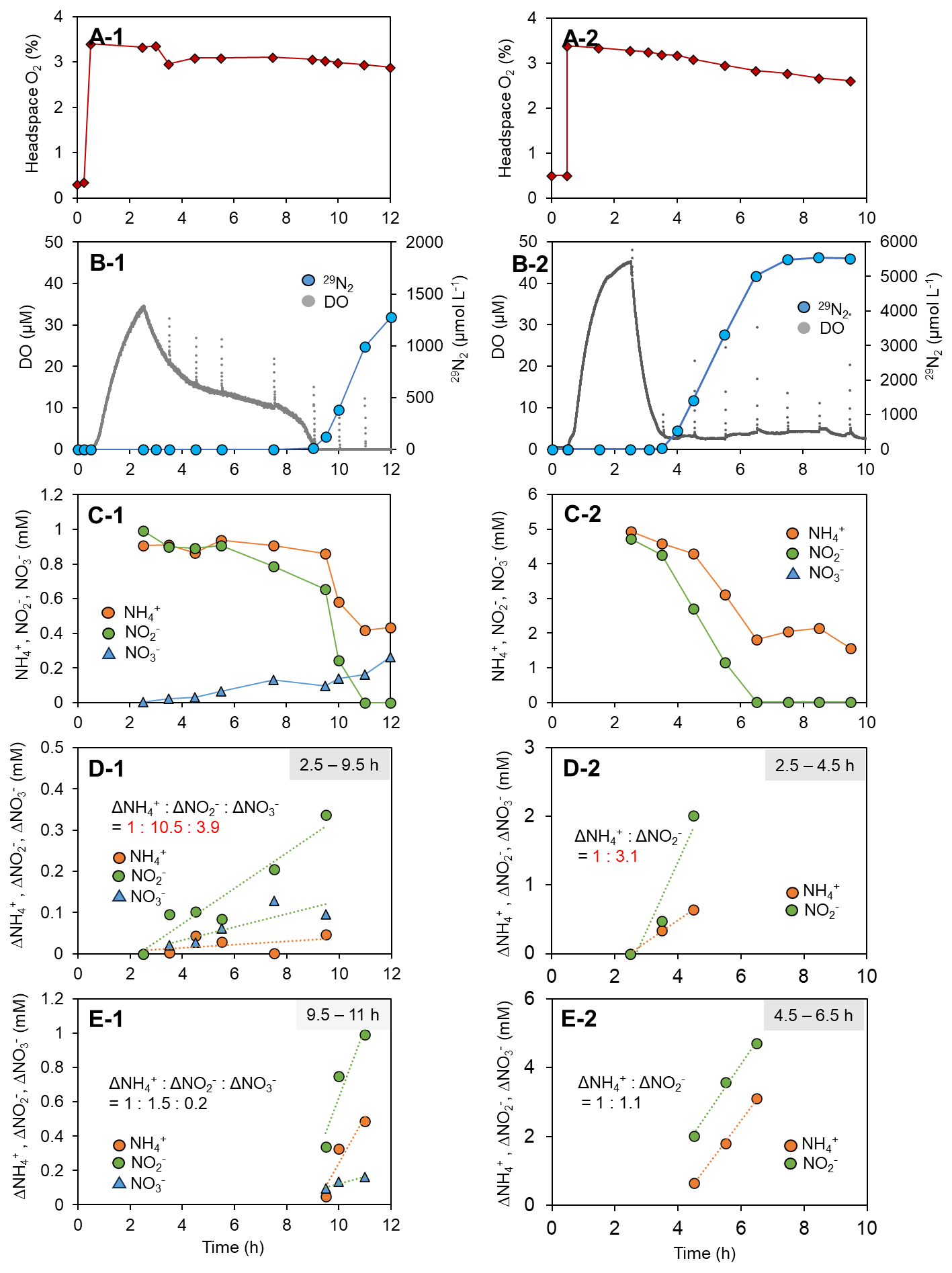
**

**Figure S7. Oxygen consumption dynamics following NH_4_^+^ and NO₂⁻ addition.**

Panels depict time-dependent changes in (**A**) headspace O₂ (%), (**B**) DO and ²⁹N₂ production, (**C**) concentrations of NH_4_^+^, NO₂⁻ and NO₃⁻, and (**D**, **E**) corresponding amounts of NH₄⁺ consumed (ΔNH₄⁺), NO₂⁻ consumed (ΔNO₂⁻), and NO₃⁻ produced (ΔNO₃⁻) before and after DO depletion, respectively. Data from two of three independent replicates are presented as **–1** and **–2**.

**
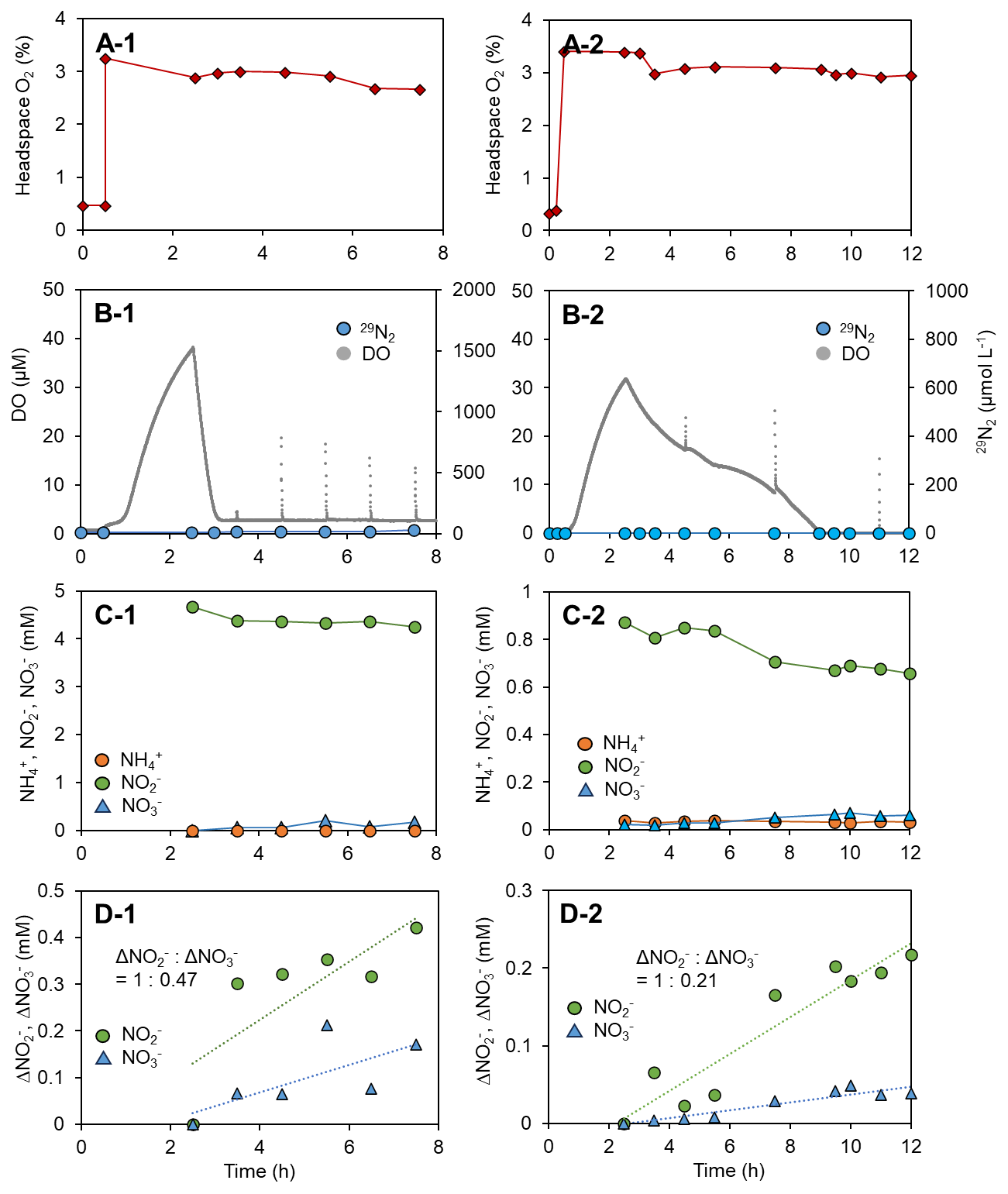
**

**Figure S8. Oxygen consumption dynamics following only NO₂⁻ addition.**

Panels depict time-dependent changes in (**A**) headspace O₂ (%), (**B**) DO and ²⁹N₂ production, (**C**) concentrations of NO₂⁻ and NO₃⁻, and (**D**, **E**) corresponding amounts of NO₂⁻ consumed (ΔNO₂⁻) and NO₃⁻ produced (ΔNO₃⁻) before and after DO depletion, respectively. Data from two of three independent replicates are presented as **–1** and **–2**.

**
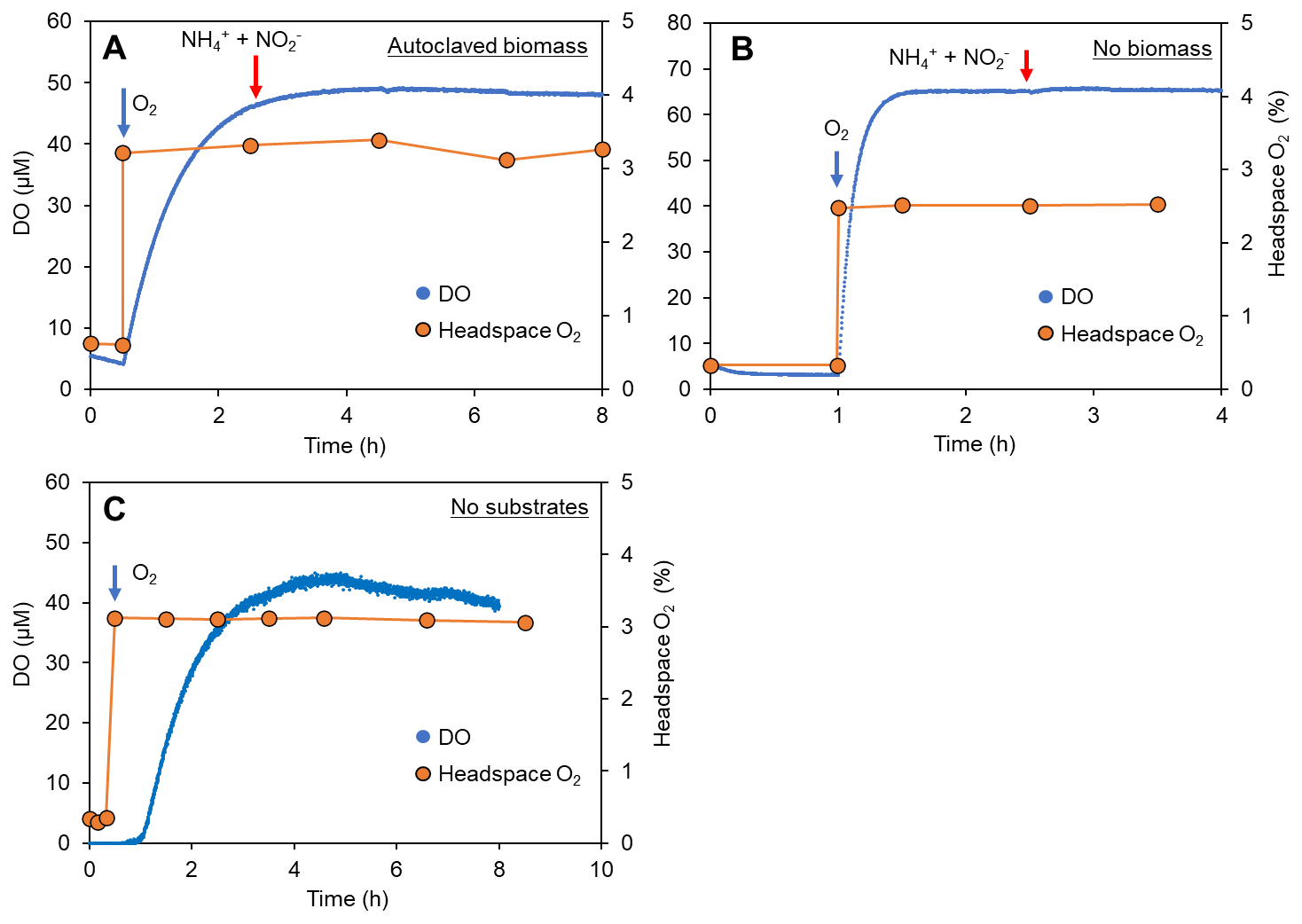
**

**Figure S9. Oxygen consumption dynamics following NH_4_^+^ and NO₂⁻ addition.**

Time-dependent changes in headspace O₂ concentration (%) and dissolved oxygen (DO) are shown under three control conditions: (**A**) with autoclaved biomass, (**B**) in the absence of biomass, and (**C**) in the absence of substrate. The absence of significant oxygen consumption across all experiments confirms that oxygen uptake is mediated by microbial activity.


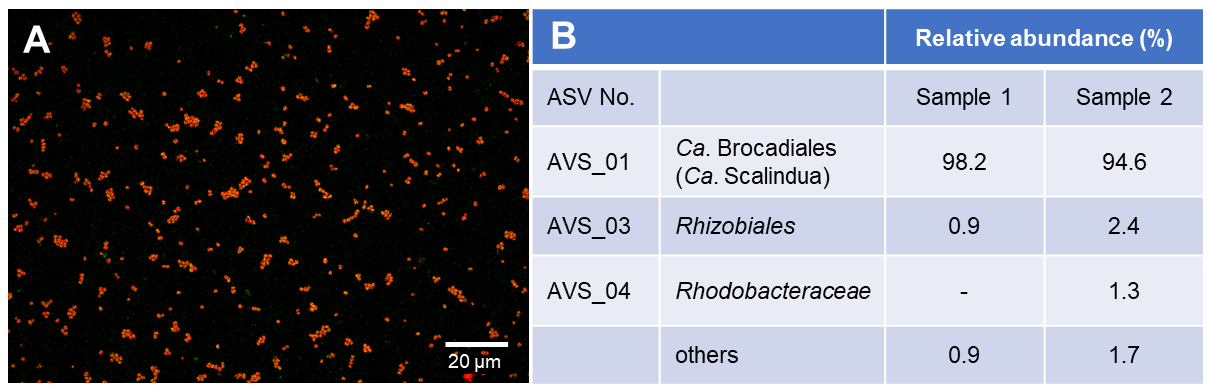


**Figure S10. Fluorescence *in situ* hybridization (FISH) image and microbial community composition of enriched *Scalindua* sp. biomass.** (**A**) Cells were hybridized with a FITC-labeled EUB338 probe mixture targeting most *Bacteria* (green) and a TRITC-labeled Scal1129b probe specific to *Scalindua* sp. (red). (**B**) Relative abundance (%) of 16S rRNA gene reads at the order level in two membrane bioreactor (MBR) samples. Amplicon sequence variants (AVSs) with < 0.5% abundance were grouped into “Others”.


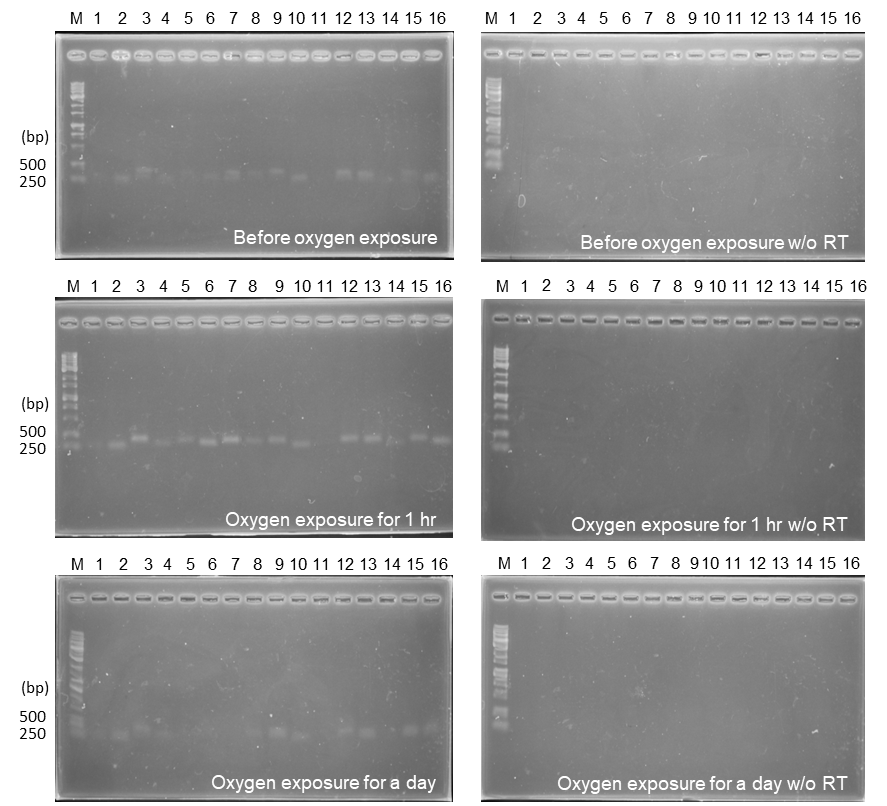


**Figure S11.** Electrophoretic profiles of amplified cDNA obtained prior to oxygen exposure, and at 1 hour and 1 day post-exposure. Distinct bands were consistently detected, whereas no amplification was observed in non-reverse-transcribed (RT) controls, confirming the absence of genomic DNA contamination and validating the RT-qPCR results.
